# Adipocytes promote breast tumorigenesis through TAZ-dependent secretion of Resistin

**DOI:** 10.1073/pnas.2005950117

**Published:** 2020-12-14

**Authors:** Yuhao Gao, Xiaosong Chen, Qing He, Ryan C. Gimple, Yajin Liao, Liang Wang, Rong Wu, Qi Xie, Jeremy N. Rich, Kunwei Shen, Zengqiang Yuan

**Affiliations:** ^a^The Brain Science Center, Beijing Institute of Basic Medical Sciences, 100850 Beijing, China;; ^b^Comprehensive Breast Health Center, Ruijin Hospital, Shanghai Jiao Tong University School of Medicine, Shanghai 200025, China;; ^c^Department of Biochemistry and Molecular and Cell Biology, Shanghai Jiao Tong University School of Medicine, Shanghai 200025, China;; ^d^Department of Medicine, Division of Regenerative Medicine, University of California, San Diego, La Jolla, CA 92037;; ^e^Department of Pathology, Case Western Reserve University, Cleveland, OH 44120;; ^f^CAS Key Laboratory of Pathogenic Microbiology and Immunology, Institute of Microbiology, Center for Influenza Research and Early-Warning (CASCIRE), Chinese Academy of Sciences, 100101 Beijing, China;; ^g^School of Life Sciences, Westlake University, 310024 Hangzhou, Zhejiang, China;; ^h^Department of Neurosciences, University of California, San Diego, School of Medicine, La Jolla, CA 92037

**Keywords:** TAZ, Resistin, adipocyte, breast tumorigenesis

## Abstract

Adipocytes have been implicated in breast tumor growth and stemness maintenance through secreted factors. However, the mechanisms by which these cytokines are regulated during diet-induced obesity and contribute to breast tumorigenesis remain largely unknown. Here we show that transcription cofactor TAZ in adipocytes is directly up-regulated by the free fatty acid/PPARγ axis upon dietary fat stimulation. TAZ knockdown alters the expression profile of a series of secreted proteins and attenuates the tumor-supporting function of adipocytes. Moreover, we identify Resistin, an adipose-derived hormone, as a functional downstream target of TAZ, which facilitates tumorigenesis, and its expression correlated with adipocyitc TAZ in triple-negative breast cancer samples. Further, Adiponectin-cre–mediated TAZ knockout in adipocytes mitigates breast tumor growth. Taken together, our findings highlight how diet-induced TAZ expression in adipocytes promotes tumorigenesis, suggesting promising cancer therapeutic targets.

## Significance

Adipocytes are the most abundant and perhaps most active components of the tumor microenvironment in obese individuals that potentiate breast tumorigenesis through secretory mechanisms. The modulation of adipocytes can be novel therapy targets for breast cancer. Here, we revealed a specific upregulation of adipocytic TAZ through the FFA/PPARγ axis in diet-induced adiposity. Adipocytic TAZ knockdown or deficiency in mice inhibits adipocyte-induced breast cancer proliferation and stemness through impaired expression and secretion of Resistin. Immunostaining in triple-negative breast cancer samples showed that higher adipocytic TAZ/Resistin expression associates with higher clinical stages and poorer survival, demonstrating promising therapeutic targets.

Breast cancer is the most common cancer and second leading cause of cancer death among women worldwide (1). Based on molecular characteristics, breast cancer is categorized into five subtypes, luminal A, luminal B/HER2-negative, luminal B/HER2-positive, HER2-positive, or triple-negative (TNBC). Compared to other molecular subtypes, TNBC is highly aggressive, lacks specific therapeutic targets, and resists chemotherapy (2).

Obesity has been proposed to be a highly significant risk factor for breast carcinogenesis, cancer progression, and aggressiveness (3), as excessive adipose tissue in obese patients not only alters the local microenvironment by remodeling extracellular matrix (ECM) but also provides both energy and niches for cancer cell proliferation, metastasis, and avoidance of chemotherapy (4, 5). Besides its inherent functions, adipose tissue is also an endocrine organ. Adipocyte hypertrophy and hyperplasia in obese individuals boost the expression of inflammatory factors, chemokines and adipokines that lead to breast cancer initiation and progression (6, 7). Among them, Resistin, an adipokine related to obesity and type 2 diabetes (8), has been investigated to promote cancer cell proliferation and migration through activating intrinsic signaling pathways, like the PI3K-AKT and MAPK pathways (9, 10). Recently, it has been reported that Resistin can also confer resistance to chemotherapy in breast cancer cells (11). Despite this, how adipocytes respond to the stimulation of excessive fat and thus initiate downstream cytokine reaction in breast cancer progression remains largely unknown.

The transcription cofactor WW domain-containing transcription regulator protein 1 (WWTR1), also known as TAZ, is the key component of the Hippo signaling pathway and regulates organ size, tissue homeostasis, mechanical stress response, and tumorigenesis (12). TAZ knockout mice display development defects including polycystic kidney disease, emphysema, and decreased adipocyte size (13, 14). In addition, TAZ is reported to modulate adipogenesis in vitro (15). Here, we show a role of TAZ in mature adipocytes regulating Resistin expression and secretion, which in turn promotes breast cancer cell proliferation and maintenance of stemness. Our findings further implicate the TAZ/Resistin network as potential chemotherapeutic targets for breast cancer treatment.

## Results

### Obesity Promotes Breast Tumor Growth and Gene Profile Variation in MAT.

To investigate the contribution of obesity to breast cancer progression, we analyzed tumor size (T stage) and lymph node involvement (N stage) status from 1,706 breast cancer patients of different molecular subtypes. Patients were segregated by body mass index (BMI) into normal weight (BMI <25), overweight (30 ≥ BMI ≥ 25), and obese (BMI >30). Obesity was associated with advanced T stages of the triple-negative and luminal A subtypes, but showed no significant correlation with other subtypes or N stages (*SI Appendix*, Fig. S1).

To directly investigate the effects of obesity on breast cancer progression, first, we fed female obesity-prone C57BL/6J mice with high-fat diet (HFD, 60% kcal) or regular chow diet (CD, 10% kcal) for 12 wk and observed that HFD induced obvious obese phenotypes, including increased body weight (*SI Appendix*, Fig. S2*A*), significant fat gain (*SI Appendix*, Fig. S2*B*), and impaired glucose tolerance (*SI Appendix*, Fig. S2*C*) in female mice, which was consistent with previous studies (16). Then, we established orthotopic allograft model by transplanting breast cancer cell line E0771 into thoracic mammary fat pad (*SI Appendix*, Fig. S2*D*). As reported (16, 17), HFD-induced obesity significantly accelerated the growth of E0771 tumors as measured by increased tumor volume and weight (*SI Appendix*, Fig. S2 *E* and *F*).

Adipocytes are active secretory cells producing various adipokines, and function largely depends on secretion in breast tumorigenesis, supporting a connection between fat accumulation and tumor cell proliferation. Therefore, we divided the established breast tumor into three areas, periadipocyte, periphery (no adipocyte), and interior, and found that cancer cells located in periadipocyte areas were more proliferative (measured by Ki67 expression) in the HFD group than in the CD group (*SI Appendix*, Fig. S2 *G* and *H*). However, cancer cell proliferation in periphery and interior areas did not vary between groups. These data confirm that obesity contributes to transplanted E0771 tumor growth and adipocytes might play an important role in breast cancer cell proliferation.

To identify potential oncogenic molecular mechanisms from obese adipose tissue, we interrogated genome-wide differential expression by RNA sequencing (RNA-seq) of mammary adipose tissue (MAT) from CD or HFD tumor-bearing mice. Kyoto Encyclopedia of Genes and Genomes analysis revealed that HFD induces notable changes in secreted protein-related pathways (*SI Appendix*, Fig. S2*I*). Among secreted protein profile, the HFD group expressed high levels of ECM proteins, adipokines, inflammatory factors/chemokines, and other proteins (*SI Appendix*, Fig. S2*J*), which was confirmed by RT-qPCR analysis (*SI Appendix*, Fig. S2*K*). Gene set enrichment analysis showed that among the top three enriched signatures, the increased expression of nearly half of the genes (41/92) has been reported in obesity-related syndromes (*SI Appendix*, Table S1). TAZ, encoding a transcription cofactor, was markedly elevated, which was confirmed by RT-qPCR analysis (*SI Appendix*, Fig. S2*L*). Collectively, these results support the induction of specific transcriptional programs in obese breast cancer hosts.

### Diet-Induced Adiposity Enhances TAZ Expression in MAT through the Free Fatty Acid/PPARγ Axis.

**Fig. 1.**
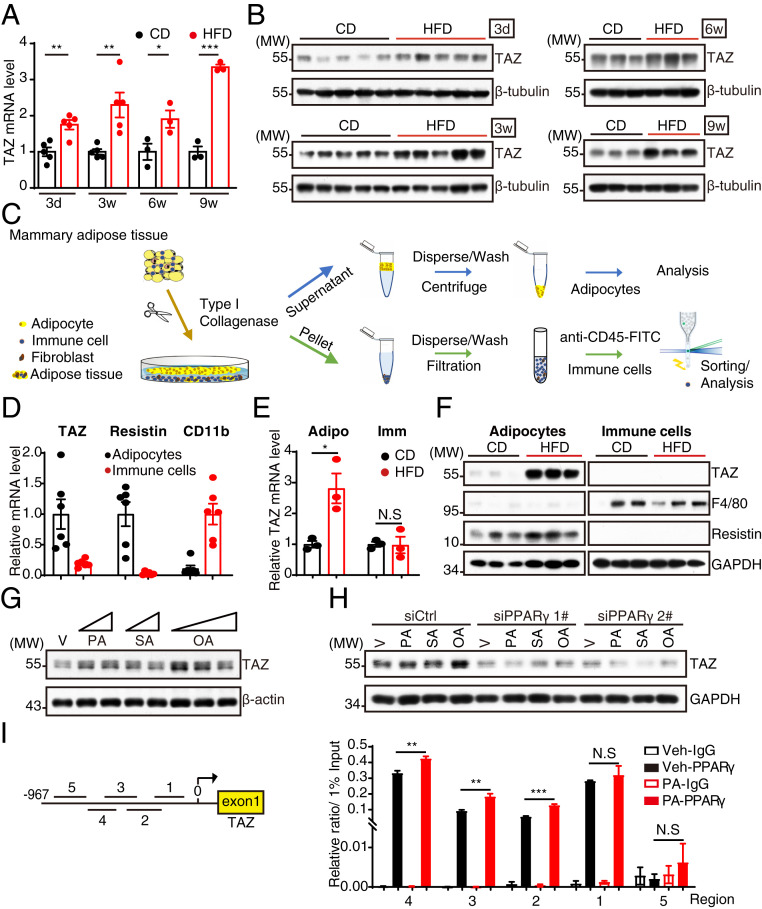
Diet-induced adiposity induces TAZ expression in adipocytes through the FFA/PPARγ axis. (*A* and *B*) Analysis of TAZ expression in MAT fed with CD or HFD for indicated times by RT-qPCR (*A*) and Western blot (*B*). (*C*) Trial schematic for isolation of mature adipocytes and immune cells from MAT. (*D*) RT-qPCR analysis of TAZ, Resistin, and CD11b expression in adipocytes and immune cells isolated from MAT. (*E* and *F*) RT-qPCR (*E*) and Western blot (*F*) analysis of gene or protein expression in adipocytes and immune cells isolated from MAT after feeding CD or HFD for 1 wk. (*G*) The 3T3-L1 adipocytes were treated with FFAs (PA, 100 and 200 μM; SA, 100 and 200 μM; OA, 100, 200, and 400 μM) or vehicle for 12 h and then harvested for Western blot analysis. (*H*) The 3T3-L1 adipocytes were transfected with small interfering RNA (siRNA) targeting PPARγ; 48 h later, the cells were treated with 400 μM FFAs for another 12 h. The cells were then harvested for Western blot analysis. (*I*) The 3T3-L1 adipocytes were treated with 400 μM palmitic acid or vehicle for 12 h and then lysed for ChIP analysis with antibody against PPARγ. Five regions from −967 ∼ −39 bp (base pair) were examined by RT-qPCR for PPARγ binding affinity. MW, molecular weight; Adipo, adipocyte; Imm, immune cell; V and Veh, vehicle. Data shown are mean ± SEM. Data were analyzed using Student’s *t* test (*A* and *E*) and two-way ANOVA (*I*). N.S.: no significance, *P < 0.05, **P < 0.01, ***P < 0.001.

As TAZ knockout mice exhibited developmental defects and decreased adipocyte size (13, 14), we speculate that TAZ may regulate the function of adipocytes in obesity-induced breast cancer progression. Intriguingly, TAZ messenger RNA (mRNA) and protein levels in MAT were induced and maintained high levels in HFD mice (Fig. 1 *A* and *B* and *SI Appendix*, Fig. S3*A*). To distinguish the expression of TAZ in MAT, we isolated primary adipocytes and immune cells from MAT, which are the two most abundant cell types within MAT (18) (Fig. 1*C*), and found that TAZ was abundantly expressed in adipocytes but not immune cells (Fig. 1*D*). Consistently, TAZ mRNA and protein levels are notably elevated in adipocytes not in immune cells upon HFD stimulation (Fig. 1 *E* and *F*). We costained TAZ with Perilipin 1, a putative adipocyte marker, in CD/HFD mice MAT and also observed increased TAZ expression in mature adipocytes upon HFD (*SI Appendix*, Fig. S3*B*). Interestingly, we observed that YAP, the paralog of TAZ, was not induced by HFD in MAT (*SI Appendix*, Fig. S3*C*). Taken together, these data above indicate that TAZ is up-regulated in adipocytes upon HFD. Blood lipids increase dramatically after HFD (19–21). Free fatty acids (FFAs) regulate the expression of genes involved in lipid and lipoprotein metabolism. Here, we also found that serum fatty acid levels were increased following administration of HFD for 3 d (*SI Appendix*, Fig. S3*D*), suggesting that FFAs may regulate TAZ expression in adipocytes. By incubating with saturated (palmitic acid [PA] and stearic acid [SA]) or unsaturated fatty acids (oleic acid [OA]), we found that TAZ mRNA and protein levels were increased in 3T3-L1 adipocytes (Fig. 1*G* and *SI Appendix*, Fig. S3*E*). FFAs regulate genes important in cell differentiation and various metabolic processes through direct interaction with peroxisome proliferator-activated receptor gamma (PPARγ) in adipocytes (22). By utilizing the dual luciferase reporter system in HEK 293T cells, we found that PPARγ directly transactivated TAZ promoter and enhanced PA-induced TAZ promoter activation in vitro (*SI Appendix*, Fig. S3*F*). In vivo administration of PPARγ ligand also confirmed the up-regulation of TAZ in MAT (*SI Appendix*, Fig. S3*G*). In order to confirm the dependency of PPARγ, we knocked down PPARγ expression in 3T3-L1 adipocytes and observed a dramatic decrease of TAZ expression and an inhibition of FFA-induced TAZ up-regulation (Fig. 1*H* and *SI Appendix*, Fig. S3*H*). Similarly, T0070907, a potent PPARγ inhibitor, blocked FFA-induced adipocytic TAZ up-regulation (*SI Appendix*, Fig. S3*I*). Furthermore, the binding affinity of PPARγ on TAZ promoter was validated by chromatin immunoprecipitation followed by PCR (ChIP-PCR), an effect enhanced by PA treatment (Fig. 1*I*). Taken together, FFAs increased TAZ expression through PPARγ-mediated gene transcription.

### Adipocytic TAZ Is Critical to Promote Breast Cancer Proliferation and Stemness.

**Fig. 2.**
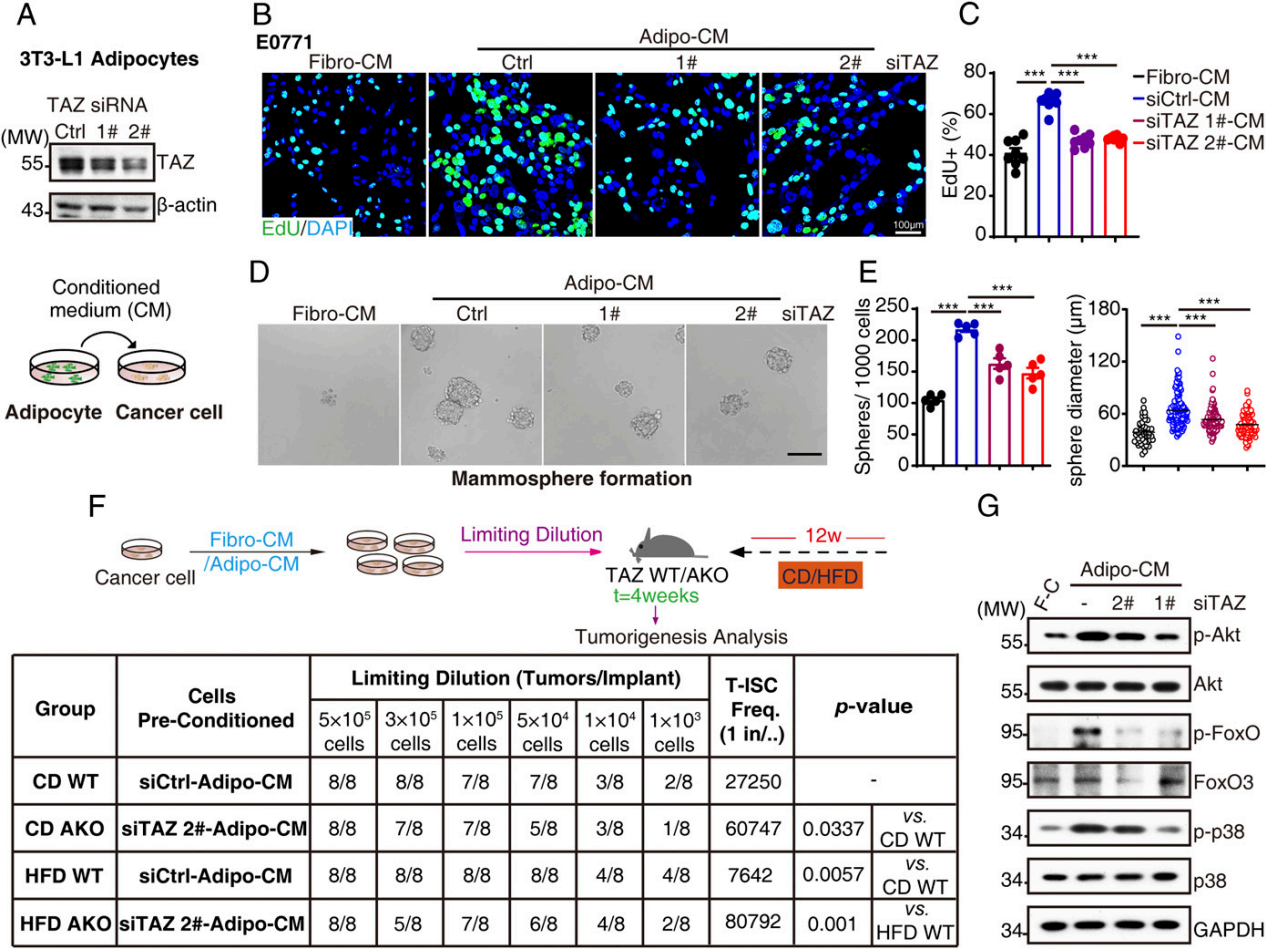
Adipocytic TAZ promotes breast cancer proliferation and stemness. (*A*) The 3T3-L1 adipocytes were transfected with siRNA targeting TAZ; 72 h after transfection, TAZ knockdown efficiency was analyzed by Western blot (*Top*); schematic representation of Adipo-CM and breast cancer cell coculture assay (*Bottom*). (*B* and *C*) E0771 cells were cultured in control or TAZ knockdown–Adipo-CM for 72 h and subjected to EdU incorporation assays. The new generation cells were detected via EdU (green). 4′,6-diamidino-2-phenylindole (DAPI)-stained nuclei are in blue. Merged view of EdU (green) and DAPI (blue) showing the overlap (*B*); the quantification for EdU staining was shown (*C*). (*D* and *E*) One thousand E0771 cells were suspension-cultured in mammosphere formation medium containing control or TAZ knockdown–Adipo-CM or Fibro-CM for 10 d; the representative images were obtained by microscopy (scale bar, 100 μm) (*D*). Mammosphere numbers were counted, and the diameter of mammosphere was measured by Image J software and shown (*E*). (*F*) E0771 cells were washed, incubated with control or TAZ knockdown–Adipo-CM for 30 min, and then harvested for Western blot analysis with indicated antibodies. (*G*) Trial schematic for breast cancer cell culture with different CMs and strategies for limit dilution analysis (*Top*). Eight weeks after culture with specified CM, E0771 cells were injected into TAZ WT or AKO mice mammary fat pad with indicated numbers (limit dilution); 4 wk after injection, stem cell frequency was determined (*n* = 8). F-C for Fibro-CM, A-C for Adipo-CM, T-ISC for tumor-initiating stem cell (*Bottom*). Data shown are mean ± SEM. Data were analyzed using one-way ANOVA. ***P < 0.001.

To address the role of adipocytic TAZ in tumor-promoting effects in vitro, we cultured breast cancer cells with conditioned medium (CM) from 3T3-L1 adipocytes (Adipo-CM, Fig. 2*A*). Compared to 3T3-L1 fibroblast-CM (Fibro-CM), Adipo-CM significantly increased E0771 and 4T1 cell proliferation as measured by EdU incorporation assay, crystal violet staining, and intracellular ATP levels, while TAZ knockdown–Adipo-CM diminished the tumor-promoting effects (Fig. 2 *B* and *C* and *SI Appendix*, Fig. S4 *A*–*C*). Critically, we observed no obvious influence of adipocytic TAZ knockdown on adipocyte differentiation measured by Oil-Red O staining and perilipin 1 expression (*SI Appendix*, Fig. S4*D*) or breast cancer cell toxicity in the CM system measured by CCK assay (*SI Appendix*, Fig. S4*E*), ruling out the effects of adipogenesis and breast cancer apoptosis. In addition to facilitating proliferation, adipocytes also promote cancer cell stemness (6); we further interrogated adipocytic TAZ in cancer stemness maintenance by mammosphere formation assay, a surrogate marker of stem cell self-renewal, and found that Adipo-CM increased mammosphere formation in E0771 cells, which was reduced by adipocytic TAZ knockdown both from sphere number and diameter (Fig. 2 *D* and *E*). Accordingly, we also found that FFA-pretreated–Adipo-CM enhanced mammosphere formation ability, while T0070907-inhibited FFA-pretreated–Adipo-CM induced mammosphere formation (*SI Appendix*, Fig. S4*F*), indicating the involvement of FFA–adipocytic PPARγ in the regulation of breast cancer cell stemness. Critically, in vivo breast tumorigenesis assay by limiting dilutions showed that Adipo-CM preconditioning significantly promoted breast tumorigenesis in HFD mice. Moreover, TAZ knockdown–Adipo-CM–preconditioned E0771 cells conferred a reduced tumorigenesis activity in TAZ AKO mice regardless of CD or HFD treatment (Fig. 2*F*), demonstrating the essential role of adipocytic TAZ in breast tumorigenesis.

Adipocyte-derived factors bind to receptors on cancer cells and initiate intracellular signaling pathways, especially PI3K-AKT and MAPK pathways, to promote cell survival (23), proliferation (24, 25), and mammosphere formation (26, 27).We observed elevated phosphorylation levels of Akt, FoxO, and p38 MAPK in breast cancer cells when exposed to Adipo-CM and reduced activation upon adipocytic TAZ silencing (Fig. 2*G*).

**Fig. 3.**
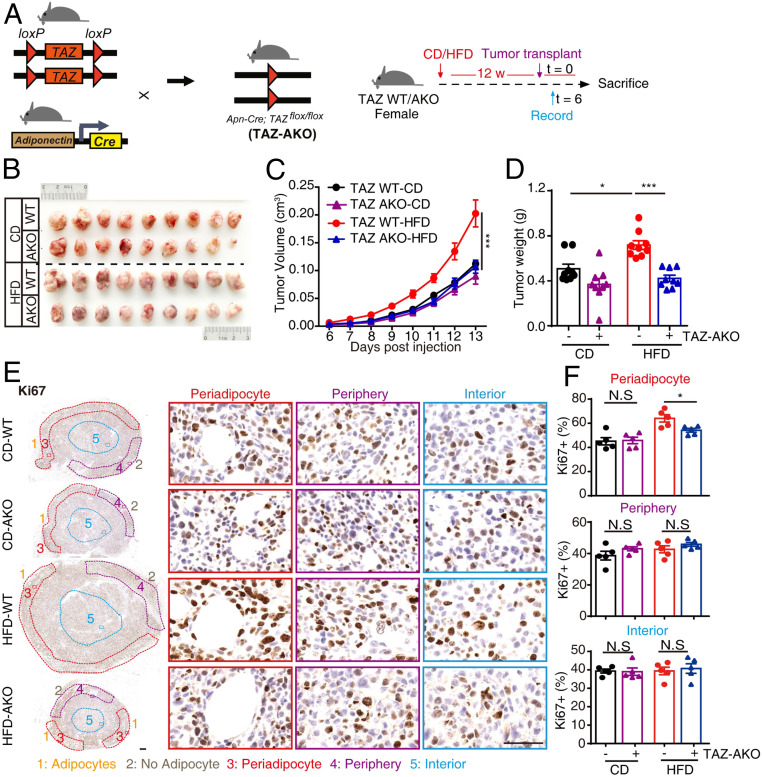
Adipocytic TAZ facilitates breast tumor growth. (*A*–*F*) The effects of adipocytic TAZ knockout on breast tumor growth. (*A*) Trial schematic for generation of homozygous Adiponectin-cre; *TAZ*^*flox/flox*^ (TAZ AKO) mice (*Left*) and strategies for mice feeding followed with breast tumor transplant (*Right*). (*B*) Representative image of breast tumors from TAZ WT and AKO mice in CD and HFD group. (*C*) Tumor volumes in each group were measured since palpable at indicated times. Tumor progressions are presented. *n* = 9 per group. (*D*) Mice were euthanized at day 13, breast tumor was resected, and tumor weight was measured. (*E* and *F*) Immunohistochemistry staining of Ki67 was performed on tumor slices; representative images of periadipocyte areas, periphery areas, and interior areas were shown (scale bar, 1 mm [*Left*] and 50 μm [*Right*]) (*E*). Ki67+ cells in each area were quantified (*F*). *n* = 5 in each group. Data shown are mean ± SEM. Data were analyzed using two-way ANOVA. N.S.: no significance, *P < 0.05, ***P < 0.001.

To explore the function of adipocytic TAZ tumor regulation in vivo, we generated adipocytic TAZ knockout mice (TAZ AKO) by crossing *TAZ*^*flox*^ mice with mice expressing cre recombinase under the control of the *adiponectin* gene promoter (Adiponectin-cre; *TAZ*^*flox/flox*^), then performed tumor transplant assays (Fig. 3*A*). The knockout efficiency in MAT was confirmed by RT-qPCR (*SI Appendix*, Fig. S5*A*). Metabolic profiles, including body weight, fat weight, lean weight, blood glucose levels, glucose/insulin tolerance, and adipocyte morphology within MAT were not obviously altered between TAZ wild-type (WT) and AKO mice upon 12 wk CD or HFD (*SI Appendix*, Fig. S5 *B*–*H*), indicating the consistent host status for breast tumorigenesis between TAZ WT and AKO mice.

Intriguingly, we found that adipocytic TAZ knockout dramatically mitigated HFD-induced tumor growth to CD levels in tumor orthotopic transplantation assay (Fig. 3 *B*–*D*). Concordantly, tumor cell proliferation in periadipocyte areas was significantly reduced in HFD-fed–TAZ AKO mice in contrast to periphery and interior areas, confirming an adipocyte specificity (Fig. 3 *E* and *F*). Taken together, these data demonstrate that adipocytic TAZ governs adipocyte-mediated breast cancer proliferation, mammosphere formation, and tumorigenesis.

### Resistin Is a Direct Target of the FFA/PPARγ/TAZ Axis.

**Fig. 4.**
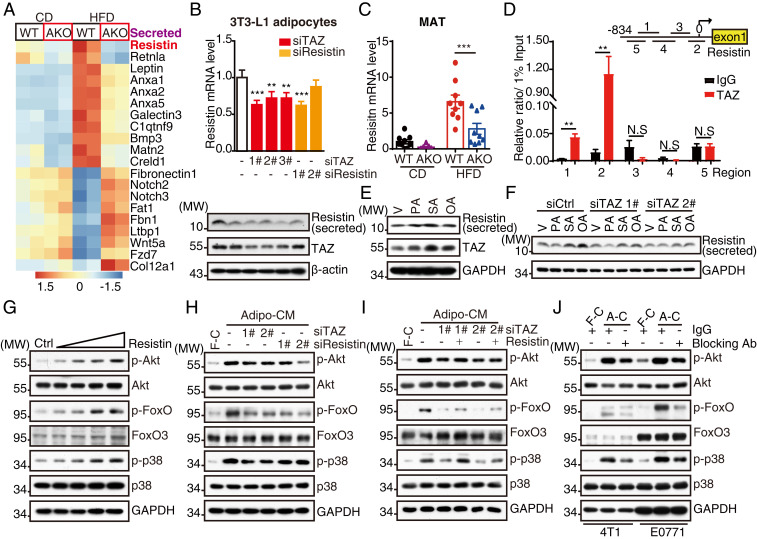
Resistin is a direct target of the PPARγ/TAZ axis in adipocytes. (*A*) Heatmap representing differential expressed genes that encode secreted proteins between TAZ WT and AKO mice MAT from CD and HFD group. (*B*) The 3T3-L1 adipocytes were incubated with siRNA targeting TAZ or Resistin; 72 h after transfection, cells were harvested for RT-qPCR (*Top*) and Western blot (*Bottom*) analysis. (*C*) MATs from TAZ WT and AKO tumor-bearing mice in CD and HFD group were lysed and analyzed the mRNA levels of Resistin by RT-qPCR. (*D*) The 3T3-L1 adipocytes were lysed for ChIP analysis with antibody against TAZ. Five regions from −834 ∼ +85 bp were examined by RT-qPCR for TAZ binding affinity. (*E*) The 3T3-L1 adipocytes were treated with FFAs (PA, 400 μM; SA, 400 μM; OA, 400 μM) or vehicle for 16 h and then harvested for Western blot analysis. (*F*) The 3T3-L1 adipocytes were incubated with siRNA targeting TAZ; 60 h after transfection, cells were treated with FFAs (PA, 400 μM; SA, 400 μM; OA, 400 μM) or vehicle in serum-free medium for 16 h. The supernatant was collected and precipitated, and cells were harvested for Western blot analysis. (*G*) E0771 cells were washed and incubated with vehicle or 10, 100, 500, 1000 ng/mL Resistin for 30 min and then harvested for Western blot analysis. (*H*) E0771 cells were incubated with control or TAZ/Resistin knockdown–Adipo-CM for 30 min and then harvested for Western blot analysis. (*I*) E0771 cells were incubated with control or TAZ knockdown–Adipo-CM supplemented with 1 μg/mL Resistin or vehicle for 30 min and then harvested for Western blot analysis. (*J*) Adipo-CM or Fibro-CM was incubated with Resistin-neutralization antibody or IgG for 12 h at 4 °C and then incubated with E0771 cells for 30 min. Cells were then harvested for Western blot analysis. Data shown are mean ± SEM. Data were analyzed using Student’s *t* test (*D*), one-way ANOVA (*B*), and two-way ANOVA (*C*). N.S.: no significance, **P < 0.01, ***P < 0.001.

**Fig. 5.**
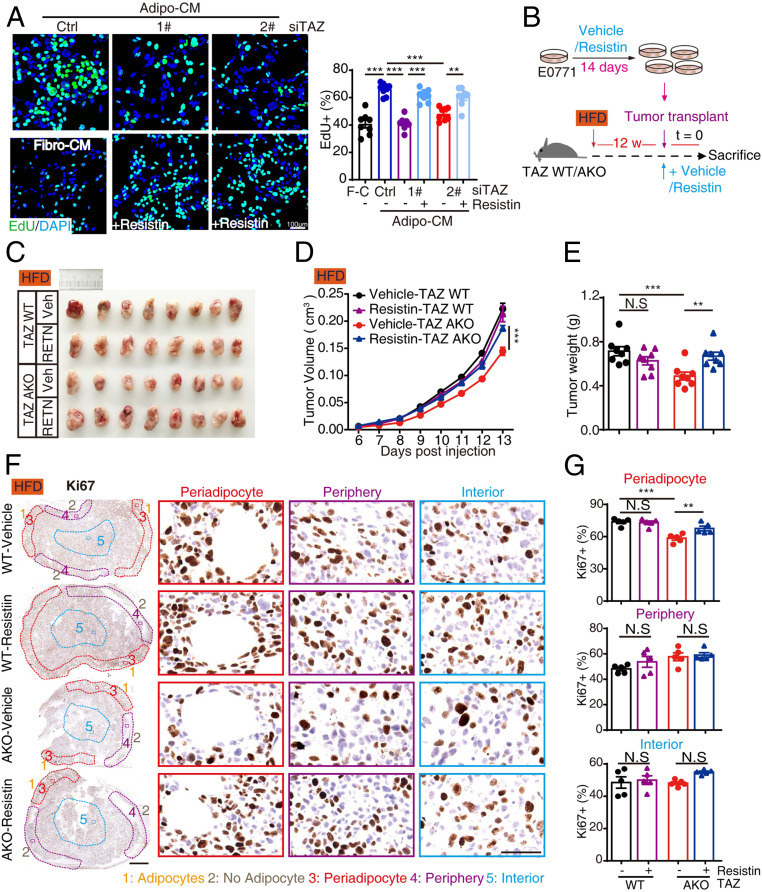
Resistin promotes breast tumor growth. (*A*) E0771 cells were cultured in control or TAZ knockdown–Adipo-CM plus 100 ng/mL Resistin or vehicle and then subjected to EdU incorporation assays. The new generation cells were detected via EdU (green). DAPI-stained nuclei in blue. Merged view of EdU (green) and DAPI (blue) showing the overlap (*Left*); the quantification for EdU staining was shown (*Right*). (*B*–*G*) The effects of Resistin on breast tumorigenesis. (*B*) Trial schematic for Resistin treatment on E0771 cells and strategies for mice feeding followed with breast tumor transplant. Briefly, E0771 cells were cultured with 50 ng/mL Resistin or vehicle for 14 d and then orthotopically injected into the fat pad of HFD TAZ WT or AKO mice supplemented with 1 μg/mL Resistin; tumor growth was monitored. (*C*) Representative image of breast tumors from vehicle and Resistin-treated group in TAZ WT and AKO mice. (*D*) Tumor volumes in each group were measured since palpable at indicated times. Tumor progressions are presented. *n* = 8 per group. (*E*) Mice were euthanized at day 13, breast tumor was resected, and tumor weight was measured. (*F* and *G*) Immunohistochemistry staining of Ki67 was performed on tumor slices; representative images of periadipocyte areas, periphery areas, and interior areas were shown (*F*). Ki67+ cells in each area were quantified (*G*). (Scale bar, 1 mm [*Left*] and 50 μm [*Right*].) *n* = 5 in each group. Data shown are mean ± SEM. Data were analyzed using one-way ANOVA (*A*) and two-way ANOVA (*D*, *E*, and *G*). N.S.: no significance, **P < 0.01, ***P < 0.001.

To gain an insight into how adipocytic TAZ supports breast cancer, we interrogated differential expression of secreted proteins in MAT derived from TAZ AKO vs. WT mice and observed diverse transcript alteration upon adipocytic TAZ deletion, especially those encoding adipokines (Fig. 4*A*). Mass spectrometry screening in TAZ knockdown–Adipo-CM revealed changes of several secreted proteins, which had been previously reported to correlate with cancer progression (*SI Appendix*, Fig. S6*A* and Table S2) and were further confirmed by RT-qPCR and Western blot analysis (*SI Appendix*, Fig. S6 *B* and *C*). Resistin was identified notably by both the RNA-seq and mass spectrometry studies (Fig. 5*A* and *SI Appendix*, Fig. S6 *A*–*C*). Interestingly, we found that in parallel to TAZ expression, HFD also significantly induced Resistin protein expression in MAT (*SI Appendix*, Fig. S6*D*), suggesting the potential regulatory network in adipocytes.

At the cellular level, both Resistin mRNA and secreted levels were significantly decreased upon TAZ knockdown in 3T3-L1 adipocytes (Fig. 4*B* and *SI Appendix*, Fig. S6*E*). In human beings, adipocytes also express and secrete Resistin (28, 29). We observed decreased Resistin protein levels when TAZ was knocked down in mature human adipocytes differentiated from preadipocytes (*SI Appendix*, Fig. S6*F*), indicating the consistent regulatory mechanism between mouse and human. In animal experiments, adipocytic TAZ deletion notably inhibited Resistin expression and secretion in MAT (Fig. 4*C* and *SI Appendix*, Fig. S6 *G* and *H*), suggesting the regulation of Resistin by TAZ. TAZ exerts its function mainly as a coactivator. By utilizing dual luciferase reporter system, we found that TAZ significantly activated Resistin promoter (*SI Appendix*, Fig. S7*A*). Further ChIP analysis in 3T3-L1 adipocytes revealed that TAZ exhibited a high affinity to specific regions on Resistin promoter (Fig. 4*D*). Taken together, these data demonstrate that TAZ transcriptionally up-regulates Resistin expression in adipocytes.

As the FFA/PPARγ axis up-regulated TAZ (Fig. 1 and *SI Appendix*, Fig. S3), we next investigated whether FFAs/PPARγ enhances TAZ-dependent Resistin expression. By dual luciferase reporter assay, we observed that Resistin promoter activity increased after 16 h FFA treatment (*SI Appendix*, Fig. S7*B*). Similar to the induction of TAZ expression, Resistin mRNA and secreted levels increased upon FFA treatment in 3T3-L1 adipocytes (Fig. 4*E* and *SI Appendix*, Fig. S7*C*), demonstrating coordinate up-regulation of TAZ and Resistin expression by FFA. In addition, T0070907 treatment blocked FFA-induced Resistin up-regulation in 3T3-L1 adipocytes (*SI Appendix*, Fig. S7*D*), suggesting that the FFA/PPARγ axis also regulates Resistin expression. However, TAZ knockdown reduced the levels of secreted Resistin in 3T3-L1 adipocytes and decreased the Resistin reporter activity upon FFA treatment (Fig. 4*F* and *SI Appendix*, Fig. S7*E*), demonstrating the necessity of TAZ in the regulation of Resistin expression. Taken together, these data indicate that excessive fatty acid influx regulates Resistin expression and secretion through the PPARγ/TAZ signaling pathway.

Mechanistically, adipocytic TAZ activates breast cancer intracellular signaling pathways through Resistin secretion; we next sought to explore the role of Resistin on this signaling. Recombinant Resistin treatment has a dose-dependent promoting effect on PI3K-AKT and MAPK signaling pathways in E0771 and 4T1 cells (Fig. 4*G* and *SI Appendix*, Fig. S8*A*). Adipocytic Resistin knockdown attenuated Adipo-CM–induced breast cancer intracellular signaling activation (Fig. 4*H* and *SI Appendix*, Fig. S8 *B* and *C*), which is similar to adipocytic TAZ knockdown. As a downstream target of TAZ, Resistin treatment rescued adipocytic TAZ knockdown-induced hypoactivation of breast cancer intracellular signaling (Fig. 4*I* and *SI Appendix*, Fig. S8*D*). To translate these findings into a clinically actionable approach, we targeted secreted Resistin in Adipo-CM using a neutralization antibody and observed reduced intracellular signaling activation (Fig. 4*J*). These data demonstrate Resistin as the functional target of adipocytic TAZ to activate breast cancer intracellular signaling.

### Resistin Promotes Breast Tumorigenesis In Vitro and In Vivo.

We further explored the functional relevance between TAZ and Resistin in breast tumor progression. Adipo-CM treatment increased breast cancer proliferation in vitro, whereas targeting TAZ or Resistin in adipocytes attenuated this effect (*SI Appendix*, Fig. S8*E*). Recombinant Resistin treatment rescued TAZ knockdown–Adipo-CM–induced impaired E0771/4T1 cell proliferation as measured by EdU incorporation assay and crystal violet staining (Fig. 5*A* and *SI Appendix*, Fig. S8 *F* and *G*). Furthermore, Resistin treatment dose-dependently increased the proportion of ALDH^+^ cells, a distinct breast cancer stem cell marker, along with enhanced stemness gene expression (*SI Appendix*, Fig. S9 *A* and *B*). Essentially, Resistin treatment rescued mammosphere formation efficiency and the size of spheres impaired by TAZ knockdown–Adipo-CM (*SI Appendix*, Fig. S9 *C*–*E*). These data demonstrate that Resistin functions as the downstream effector of adipocytic TAZ to promote breast tumorigenesis in vitro.

To investigate the protumoral role of Resistin in vivo, we first incubated E0771 cells with the recombinant Resistin for 14 d in vitro and then orthotopically transplanted the cells into mammary fat pads of HFD-fed TAZ WT or AKO mice (Fig. 5*B*). Consistent with the in vitro results, Resistin treatment significantly rescued adipocytic TAZ knockout-induced E0771 tumor growth retardation (Fig. 5 *C*–*E*). Concordantly, tumor cell proliferation in periadipocyte areas was significantly rescued in HFD-fed–TAZ AKO mice in contrast to periphery and interior areas, confirming the adipocyte specificity (Fig. 5 *F* and *G*), suggesting the importance of Resistin on E0771 tumor progression in vivo. To test whether neutralizing Resistin is able to block tumor progression in vivo, we orthotopically injected E0771 cells into mice pat pad after feeding with an HFD or CD for 12 wk and followed by a neutralizing antibody or control IgG administration around the tumor (*SI Appendix*, Fig. S10*A*). We observed that Resistin neutralization significantly decreased breast tumor progression with a reduced tumor volume and weight compared with the IgG group (*SI Appendix*, Fig. S10 *B*–*D*). Taken together, these data demonstrate that adipocytic TAZ/Resistin signaling facilitates breast tumorigenesis, and Resistin neutralization may be a therapeutic strategy for breast cancer treatment.

### Clinical Relevance of Adipocytic TAZ/Resistin in TNBC.

**Fig. 6.**
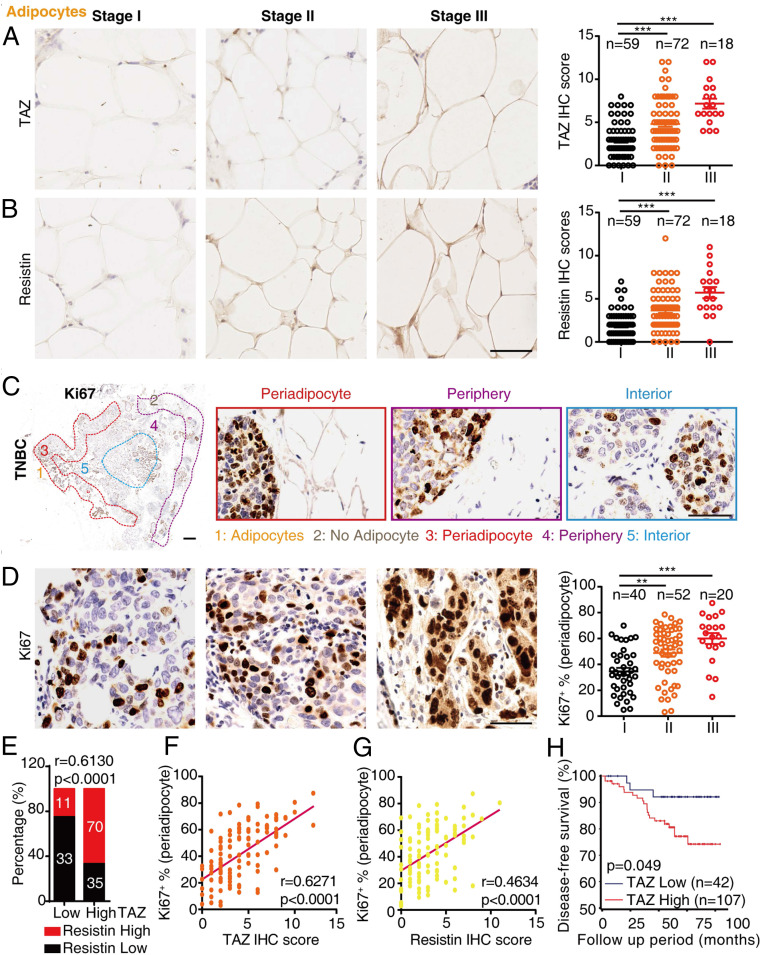
Clinical relevance of TAZ and Resistin in TNBC patient adipocytes. (*A* and *B*) Representative images showing TAZ (*A*) or Resistin (*B*) expression in TNBC patient adipocytes from different clinical stages (*Left*). The expression was assessed (*Right*). (Scale bar, 1 mm and 50 μm, *n* = 149.) (*C*) Immunohistochemistry staining of Ki67 was performed on TNBC specimens; representative images of periadipocyte areas, periphery areas, and interior areas were shown. (Scale bar, 1 mm [*Left*] and 50 μm [*Right*].) (*D*) Representative images showing Ki67 staining of breast tumor cells in periadipocyte areas from different clinical stages (*Left*). Ki67+ cells in periadipocyte areas were quantified (*Right*). (Scale bar, 1 mm and 50 μm, *n* = 112.) (*E*) The expression of TAZ or Resistin was categorized by low and high expression as described in *Materials and Methods*, and the correlation of TAZ with Resistin was analyzed. (*F* and *G*) Scatterplots of periadipocyte Ki67+ proportion related to TAZ (*F*) or Resistin (*G*) immunohistochemistry scores in patient samples (*n* = 112). (*H*) Disease-free survival related to low or high adipocytic TAZ expression was analyzed in 149 breast cancer patients. Data shown are mean ± SEM. Data were analyzed using one-way ANOVA (*A*, *B*, and *C*), Pearson’s correlation (*E*, *F*, and *G*), and log-rank test (*H*). **P < 0.01, ***P < 0.001.

We focused our next investigation of TAZ/Resistin and breast cancer on TNBC, which also showed the most significant association between obesity and clinical T stages (*SI Appendix*, Fig. S1). Immunohistochemistry analysis of TAZ and Resistin in adipocytes showed an increased expression in samples with more advanced clinical stages (Fig. 6 *A* and *B*), indicating the correlation between TAZ/Resistin expression and TNBC progression. Similar with the segregation of transplanted E0771 cells in murine samples, human TNBC samples were also divided into three areas, including periadipocyte areas, periphery areas (no adipocyte), and interior areas (Fig. 6*C*). There is a significant increase of Ki67+ cells within the periadipocyte areas, and Ki67 expression levels were positively correlated with clinical stages (Fig. 6*D*). Further analysis showed that consistent with the results from mice and cell lines, TAZ expression levels in adipocytes displayed a strong correlation with Resistin expression (Fig. 6*E*, *r* = 0.6130; *P* < 0.0001); adipocytic TAZ or Resistin expression levels also showed a significantly positive correlation with tumoral Ki67 expression within periadipocyte areas (Fig. 6*F*, *r* = 0.6271; *P* < 0.0001; Fig. 6*G*, *r* = 0.4634; *P* < 0.0001). However, we observed only a weak correlation between adipocytic TAZ or Resistin and tumoral Ki67 expression within periphery or interior areas (*SI Appendix*, Fig. S11 *A* and *B*), suggesting a close connection between adipocytic TAZ/Resistin expression and TNBC growth. In addition, we found that serum Resistin levels were elevated in TNBC patients with advanced clinical stages (*SI Appendix*, Fig. S11*C*), indicating that circulating Resistin might be associated with TNBC development. Importantly, Kaplan–Meier estimates of disease-free survival analysis in TNBC patients also showed that adipocytic TAZ expression exhibited a strong positive correlation with poor prognosis (Fig. 6*H*).

## Discussion

Breast cancer microenvironment is a heterogeneous ecosystem consisting of matrix, adipocytes, fibroblasts, and diverse immune cells. Adipocytes are the most abundant and perhaps most active components among tumor microenvironments especially in obese individuals (6, 30). Evidence implicates that interactions between breast cancer and adjacent adipocytes might promote breast cancer initiation and progression. However, systematic study on regulation and function of adipocyte-facilitated breast tumorigenesis is lacking. Here, our study establishes the FFA/PPARγ/TAZ/Resistin axis as an essential signaling pathway in adipocytes that facilitates breast cancer proliferation and stemness, with an implication of a diagnostic and therapeutic avenue for breast cancer.

The interactions between adipocytes and tumor cells have been extensively investigated. In several tumors such as breast (31), prostate (32), ovarian (33), and colon (34) cancer, elevated BMI indicates poor clinical outcomes. Dysfunctional adipocytes in obese individuals release a disturbed profile of adipokines which play distinct roles in establishing the peritumoral environment and promoting tumorigenesis via a complex adipocyte–cancer cell paracrine loop (35). Our mechanistic studies showed that diet-induced adiposity prompted adipocytic TAZ/Resistin expression and therefore enhanced breast cancer cell proliferation and stemness maintenance. Here, our study of 1,706 breast cancer patients found that obesity correlated positively with more advanced clinical stage, especially T stage in TNBC. Coincidently, adipocytic TAZ/Resistin is strongly correlated with advanced clinical stage, especially T stage, demonstrating the critical tumor-supporting role of adipocytes on TNBC.

Triple-negative breast tumors are heterogeneous, with mesenchymal-like cancer cells at the interior and rapidly proliferating cancer cells in the periphery (36, 37). Clinically, we have shown that obesity is mainly associated with breast cancer size (T stage). We found that breast cancer cells neighbored to adipocytes confer higher proliferation in both transplanted tumor blocks and human samples, indicating that adipocytes are critical for breast cancer proliferation, although we could not rule out the involvement of systemic effects in both TAZ AKO mice upon HFD treatment and human samples. In fact, an increasing number of studies have investigated targeting adipocytes to treat cancer. For example, weight loss was shown to be sufficient to reduce serum cytokine levels and thus inhibit cancer cell proliferation and subsequent lung metastasis (38).

Previous studies have reported that TAZ inhibits adipocyte differentiation through repressing the transcriptional activity of PPARγ in mesenchymal stem cells (MSCs)/preadipocytes (15). However, our study focused the function of TAZ on mature adipocytes and observed a regulation between PPARγ and TAZ. As PPARs play essential roles not only in MSCs/preadipocytes during adipogenesis (15), but also in mature adipocytes to regulate the fatty acid metabolism and adipocyte hypertrophy (39), we suggest that TAZ also plays distinct roles under the regulation of PPARγ in mature adipocytes. In order to rule out the effects of TAZ deficiency on adipocyte differentiation, we knocked down TAZ in mature adipocytes in vitro and depleted TAZ in vivo by using Adiponectin-cre, which only expressed in mature adipocytes. Our results in mature adipocytes revealed that TAZ was essential in response to the stimulation of upstream signals (FFAs/PPARγ) and the regulation of Resistin expression and secretion.

Resistin is secreted by adipocytes in mice, while in human beings, Resistin has been reported to be mainly associated with monocytes/macrophages (40, 41). However, the isolated human adipocytes also express (29) and secrete Resistin in a considerable amount (∼3.15 ng/d/500,000 cells in vitro) (28). Accordingly, by differentiating human adipocyte in vitro, we validate that human mature adipocytes express Resistin and TAZ knockdown reduced the expression, which was coincident with the cell line and murine models.

Other groups have identified the roles of Resistin in migration and chemotherapy resistance in cells (7, 42). Recently, Wang et al. have reported that Resistin promotes breast cancer metastasis and stemness through Toll-like receptor 4 (TLR4) signaling, suggesting TLR4 as a receptor for Resistin-mediated breast cancer progression (43). Here, our findings have extensively explored the function of adipocytic TAZ in Resistin regulation and the role of the TAZ/Resistin axis in breast tumorigenesis. Together, the TAZ/Resistin/TLR4 axis may serve as a link between adipocyte and nonautonomous breast cancer progression, and Resistin might be a therapeutic target for clinical breast cancer treatment.

Mechanistically, we find the expression of TAZ, a key effector of the Hippo signaling pathway, is significantly elevated in mature adipocytes under the regulation of the FFA/PPARγ axis and subsequently induces the expression and secretion of Resistin to facilitate breast tumorigenesis. Besides transcriptional regulation, TAZ is also tightly regulated by phosphorylation-induced nucleus exclusion to restrain its transcriptional activity (44). Immunohistochemistry in MAT of HFD mice also exhibits increased nuclear localization of TAZ in adipocytes, indicating that HFD enhances not only the expression level but also the transcriptional activity of TAZ in adipocytes. Interestingly, we observed that YAP, the paralog of TAZ, was not induced by HFD in MAT, indicating that TAZ is a major player of Hippo signaling in HFD-induced adiposity.

In summary, our findings demonstrate that TAZ is specifically up-regulated by FFAs/PPARγ in adipocytes upon dietary fat stimulation and facilitates breast cancer proliferation and stemness, implying a potential role of adipocytic TAZ in the pathogenesis of breast cancer. Furthermore, we propose that Resistin is a functional target gene of TAZ in adipocytes. Our data further show that TAZ and Resistin are correlated in TNBC with poor prognosis. These findings support targeting of a critical cross-talk within the tumor microenvironment as a promising therapeutic strategy for TNBC therapy.

## Materials and Methods

Details of all materials regarding list of human clinical samples, reagents and antibodies, mouse strains, primers for siRNA, RT-qPCR and ChIP analyses for our studies can be found in *SI Appendix*. Further, methods detailing cell culture and transfection, differentiation of 3T3-L1 fibroblasts and human preadipocytes, dual luciferase reporter system, FFA preparation, Flow cytometry of ALDH^+^ cells, cellular ATP determination, glucose tolerance tests and insulin tolerance tests, ELISA for human and mouse Resistin, RNA isolation and RT-qPCR, CM system and mass spectrometry, immunoneutralization, mammosphere formation assay, isolation of adipocytes and immune cells, Oil-Red O staining, immunohistochemical staining and evaluation, EdU incorporation assay, chromatin immunoprecipitation, RNA-seq and analysis, allograft experiments, and in vivo limiting dilution assay can also be found in *SI Appendix*.

All animal experimental procedures were reviewed and approved by the Institutional Animal Care and Use Committees of Beijing Institute of Basic Medical Sciences (Beijing, China). This study was approved by the internal review and ethics boards of Ruijin Hospital. Informed consent was obtained from all patients.

## Supplementary Material

Supplementary File

## Data Availability

The data reported in this paper have been deposited in the National Center for Biotechnology Information sequence read archive database with links to BioProject accession ID PRJNA562114.
