# Supplementary material for: Adipocytes promote breast tumorigenesis through TAZ-dependent secretion of Resistin
Source: Proc Natl Acad Sci U S A. 2020 Dec 14;117(52):33295–304. doi: 10.1073/pnas.2005950117 (PMC7776784; doi:10.1073/pnas.2005950117)
Supplement: Supplementary File [file pnas.2005950117.sapp.pdf]

SI Appendix: Materials and Methods

Human clinical samples

Clinical data of 1706 patients with primary breast carcinomas were collected from Comprehensive Breast Health Center, Ruijin Hospital (Shanghai Jiao Tong University School of Medicine, Shanghai, China) for BMI and T/N staging analysis, the time frame of patients enrolled was from December 13, 2012 to February 1, 2016. 149 TNBC samples were used for IHC staining and clinical outcome analysis, the time frame of these patients was from February 5, 2009 to May 22, 2017. 149 patients with TAZ detection, the mean age was 56.54 +/- 11.82 years old and the median age was 56 (28-86) years old. The mean follow-up period was 63.0 +/- 26.9 months and the median follow-up period was 66.9 (2.7-104.2) months. 78 blood samples from TNBC patients received no prior chemotherapy or radiotherapy before surgery were collected for serum Resistin determination. Normal distribution was performed using SPSS 22.0.

Reagents and antibodies

Mouse recombinant Resistin protein was purchased from PeproTech (#450-28, Rocky Hill, NJ, USA), palmitic acid (#P0500), steric acid (#S4751) and oleic acid (#O1008) were purchased from Sigma-Aldrich (St Louis, MO, USA), T0070907 (#S2871) and Rosiglitazone (#S2556) were purchased from Selleckchem (Houston, TX, USA), fatty-acid-free-BSA was from Equitech-Bio, Inc. (#BAH66, Kerrville, TX, USA). Antibodies used for immunoblotting were as follows: anti-TAZ (#4883), anti- Perilipin-1 (#3470), anti-Akt (#4691), anti-p38 MAPK (#8690), anti-phospho-Akt S473 (#4060), anti-phospho-FoxO T24/T32 (#9464), anti-phospho-p38 MAPK T180/Y182 (#9211) and anti-p-S127 YAP (#13008) were purchased from Cell Signaling Technology (Beverly, MA, USA), anti-YAP was from NOVUS Biologicals (#NB110-58358, Littleton, CO, USA), anti-Resistin (#500-P182G) was from PeproTech, anti-FoxO3 (#sc-11351, Santa Cruz, Dallas, TX, USA), anti-Galectin (#ab108389), anti-Anxa2 (#ab178677) and anti-GDIR1 (#ab133248) were from abcam (Cambridge, MA, USA), and anti-Complement factor D (#A8117) were from ABclonal Technology (Wuhan, HB, China), anti-GAPDH (#AT0002), anti-β-actin (#AT0001) and β-tubulin (#AT0003) were from CMCTAG (Milwaukee, WI, USA).
Western blotting was performed as previously described(1), when showing the western blotting bands, molecular size markers are interpolated.

Mouse strains

TAZ conditional knockout mice were generated by using the CRISPR/Cas 9 method. Briefly, the loxP elements were inserted into the intron upstream and downstream of exon 3, $TAZ^{flox/+}$ mice were positively confirmed by Southern blot. To induce adipocyte-specific genetic ablation, homozygous mice were bred to mice expressing cre recombinase driven by *adiponectin* promoter. Mice were housed in a 12 hr/12 hr light-dark cycle at 22-24°C with free access to water, for diet induced adiposity study, mice were fed with a high-fat diet (60% kcal fat, D12492, Research Diets) for indicated times.

All animal experimental procedures were reviewed and approved by the Institutional Animal Care and Use Committees of Beijing Institute of Basic Medical Sciences (Beijing, China).

Cell culture and transfection.

293T (or HEK 293T), 3T3-L1 fibroblast and 4T1 mouse cell lines were obtained from ATCC (Manassas, VA, USA). The E0771 cell line was a kind gift from Dr. Rong Xiang (NanKai University, China), human preadipocytes were a kind gift from Dr. Wanzhu Jin (Institute of Zoology, Chinese Academy of Sciences, China). HEK 293T cells were maintained in Dulbecco's modified Eagle's medium (DMEM, Invitrogen, Waltham, MA, USA) supplemented with 10% fetal bovine serum (FBS, Gibco, Grand Island, NY, USA), 3T3-L1 fibroblasts were grown in DMEM, supplemented with 10% calf serum (Kang Yuan Biology, Tianjin, China), human preadipocytes were maintained in DMEM/ F12 (Gibco), supplemented with 10% FBS. 4T1 and E0771 cells were grown in RPMI 1640 medium (Gibco), supplemented with 10% FBS. All the cell mediums were supplemented with in 1% Penicillin-Streptomycin (Invitrogen) and cells were maintained in 5% CO_2 atmosphere at 37°C.

Gene transient knockdown in 3T3-L1 adipocytes was performed by siRNA transfection with Lipofectamine® RNAiMAX Transfection Reagent (#13778150, Invitrogen). The targeting sequences of siRNAs are listed in ***SI Appendix*, Table 3**.

Differentiation of 3T3-L1 fibroblasts and Human preadipocytes

3T3-L1 fibroblast was grown to confluence. At day 0, cells were pretreated with DMEM plus 10% FBS and 2 μM insulin (#I-5500, Sigma-Aldrich) for 2 days. To begin with differentiation, the medium was changed to DMEM plus 10% FBS and 2 μM insulin, 10 μM dexamethasone (#D-4902, Sigma-Aldrich), and 0.5 mM isobutyl-methylxanthine (#I-7018, Sigma-Aldrich) for 3 days, cells were then incubated with 2 μM insulin for another 2 days. Differentiation was achieved when medium was changed to DMEM plus 10% FBS for 4 days.

For differentiation of human preadipocytes, cells were grown to confluence, and induced in medium containing 0.5 mM isobutyl-methylxanthine, 10 μM dexamethasone, 10 μM insulin, 0.2 nM triiodothyronine (T3) (#T-2752, Sigma-Aldrich) for four days and maintained in medium with 10 μM insulin for one day. The treatment was repeated three times, after which the cells were maintained in DMEM with 10 μM insulin until day 21 and subjected to oil red O staining to detect cytoplasmic triglyceride for mature adipocyte verification.

Dual luciferase reporter system

Mouse TAZ and Resistin promoter were cloned into pGL3-luciferase reporter vector (Promega, Madison, WI, USA). The luciferase reporter plasmid and pCMV-Renilla plasmid were transiently transfected into 293T cells, 6 hr after transfection, cells were treated with free fatty acids or vehicle for indicated concentration if needed, 12 or 16 hr after treatment, 293T cells were lysed and the fluorescence values were measured as the manufacturer suggested.

Free fatty acid preparation

As reported(2-4), free fatty acid stock solution was prepared by conjugating free fatty acid with fatty acid-free BSA. In detail, 100 mM free fatty acid stock solution was dissolved in 100 mM NaOH by heating at 70 °C (SA at 90°C). The stock solution was then appropriately diluted in

prewarmed 20% BSA solution (final BSA concentration 0.5% (w/v)) when use. Vehicle (control medium) contained 100 mM NaOH and BSA without lipid.

Flow cytometry of ALDH$^+$ cells

E0771 cells were washed with phosphate-buffered saline (PBS) and then detached with trypsin/(0.05% EDTA). Detached cells were washed twice with PBS containing 10% FBS (wash buffer) and resuspended in the wash buffer (10^6 cells/100 µL). For Aldefluor assay, E0771 cells were centrifuged and resuspended in Aldefluor assay buffer containing ALDH substrate (BODIPY-aminoacetaldehyde) and incubated at 37°C for 45 min. A specific inhibitor of ALDH, diethylaminobenzaldehyde (DEAB), was used as a negative control (#01700, Stem Cell Technologies, Vancouver, BC, Canada). The labeled cells were washed once and resuspended in wash buffer, and then analyzed on a FACS Vantage (BD Biosciences, San Jose, CA, USA).

Cellular ATP determination

Breast cancer cells were plated into a 96-well plate at a low density of about 10 %, 24 hr later, the supernatant was aspirated and cellular ATP level was determined using a detection kit (#G9241, Promega) as the manufacturer suggested and recorded as the hour 0, the rest cells were then washed and cultured with Fibro-CM or TAZ knockdown-Adipo-CM, every 12 hr, the cellular ATP levels were measured and recorded.

Glucose tolerance tests and insulin tolerance tests

For glucose tolerance tests (GTT), animals were fasted for 16 hr overnight (17:00 pm-09:00 am) with free access to drinking water. The glucose level was assessed following glucose injection (2.0 g/kg) intraperitoneally. Serum glucose levels were determined immediately before and 15, 30, 60 and 120 min after glucose injection using a glucometer (OneTouch Ultra, Bayer, Berlin, Germany). For insulin tolerance tests (ITT), mice were fasted for 4 hr (9:00 am-13:00 pm) and then injected with human insulin (0.75 U/kg for C57BL/6J background mice) intraperitoneally. Blood glucose levels were determined immediately before and 15, 30, 60 and 120 min after insulin injection.

ELISA for Human and mouse Resistin

Human Resistin ELISA kit was purchased from GenStar (#C630-02, Beijing, China), Mouse Resistin ELISA kit was from Invitrogen (# EMRETN). Samples were measured as the manufacturer suggested. Briefly, serum or supernatant samples were diluted with dilution buffer and added into the capture antibody precoated 96-well plates followed by incubated in 37°C or room temperature for 90 or 150 min. The plates were then washed 4 times with wash buffer for 2 min each, followed by adding biotinylated capture antibody and incubated in 37°C or room temperature for 60 min. After washing for 4 times, the plates were added with Horseradish Peroxidase working buffer in 37°C or room temperature for 30 or 45 min. Finally, each well was added with 100 µl tetramethylbenzidine substrate and incubated at room temperature for 10 min in the dark. The reaction was stopped by adding 100 µl of stop buffer to each well and the OD value was measured at 450 nm immediately by an ELISA reader (Magellan, Tecan Group

AG, Männedorf, Switzerland).

RNA isolation and RT-qPCR

Cultured cells were lysed by TRIzol™ Reagent (#15596018, Invitrogen) (adipose tissues and other tissues were cut into pieces in TRIzol and immediately mashed together until homogenous with a homogenizer), total RNA was isolated according to the manufacturer's instructions. mRNA was converted to cDNA with the cDNA synthesis kit (AE311-03, TransGen Biotech, Beijing, China). RT-qPCR was performed with diluted cDNA (1:4) in three wells for each primer and SYBR green master mix (Bio-Rad) on Bio-Rad iCycler iQ Real Time PCR system. All RT-qPCR experiments were repeated at least three independent times. Primers used are listed on ***SI Appendix*, Table S4**.

Conditioned medium system and mass spectrometry

The medium of 3T3-L1 fibroblast or 3T3-L1 adipocyte were removed, the plates were washed two times with sterile PBS. 4 mL serum-free DME containing 2% fatty acid free-BSA per 10 cm dish was added to the plate and incubated for 12 hr. After incubation, the medium was collected and centrifuged at 500 × g and filtered through a 0.22 μm filter to remove cellular debris. The conditioned medium was then incubated with 4T1 or E0771 cells for 0.5 hr to detect the changes of intrinsic signaling pathways. For crystal violet staining, cells were cultured in conditioned medium supplemented with 0.2% FBS for 48-72 hr and then fixed with 4% paraformaldehyde solution and stained with 0.2% crystal violet solution.

For mass spectrometry assay, the confluent 3T3-L1 fibroblast or TAZ siRNA transfected adipocytes in 10 cm dish were washed twice with PBS and directly cultured in 4 mL serum-free DMEM for 12 hr. After centrifugation and filtration through a 0.22 μm filter, the medium was flash-frozen in liquid nitrogen and freeze-dried for 3 days using ALPHA 1-2 LD plus freeze dryer (Christ, Osterode, Germany). The dry power from 3T3-L1 fibroblast or adipocyte was dissolved in sterile PBS at 200 mg/mL. 10 mg of each sample was loaded to SDS-PAGE gel for silver staining, and the bands under 55 kDa were subjected to mass spectrometry sequencing and data analysis. Briefly, the gels were minced and destained with 50% acetonitrile in 50 mM ammonium bicarbonate and then added 10 mM DTT to reduce proteins at 56℃, followed by alkylation with 55 mM iodoacetamide at room temperature in the dark. After that, the samples were trypsin digested overnight at 37℃ with gentle shaking. Peptides were then extracted by using 1% trifluoroacetic acid in 50% acetonitrile followed by vacuum-dried and reconstituted in 0.1% formic acid for MS analysis. The treated samples were examined by nanoLC-MS/MS (nanoACQUITY UPLC and SYNAPT G2 HD mass spectrometer, Waters). MS/MS data were obtained with Data Dependent Analysis mode and processed with PLGS 2.4 software (Waters), and the resulting peak list was searched against the NCBI database with the MASCOT search engine.

Immunoneutralization

3T3-L1 fibroblast or adipocyte conditioned medium was incubated with Resistin-neutralization antibody (PeproTech, 10 μg/mL) or the same amount of goat IgG control at 4°C overnight to

make it neutralized thoroughly by inversions, after centrifugation at 500 × g, the supernatant was placed on 4T1 or E0771 cells for 0.5 hr to detect the phosphorylation level changes of growth signaling molecules.

Mammosphere formation assay

Normal mammosphere formation medium was prepared by DMEM/F12 (Gibco) supplemented with 2% B-27 (#17504044, Invitrogen), 20 ng/mL EGF (#315-09, PeproTech), 20 ng/mL bFGF (#450-33, PeproTech), when cocultured with Fibro-CM or Adipo-CM, the mammosphere formation medium was changed to fresh and filtered Fibro-CM or Adipo-CM plus 10% normal mammosphere formation medium and 0.5% methylcellulose (#HSC001, R&D Systems, Minneapolis, MN, USA). 1000 flow-sorted E0771 cells per well were plated in Corning® Costar® ultra-low attachment 96 well plates (#CLS3474-24EA, Sigma-Aldrich) with 200 μL medium (with 1 μg/mL Resistin or vehicle), after 7-10 days, total mammosphere number was calculated and the formed spheres were photographed under inverted NIKON microscope. The diameter of sphere was measured using Image J software (NIH, USA).

Isolation of adipocytes and immune cells

Primary adipocytes and immune cells were isolated according to the published methods(5, 6). In brief, Mammary adipose tissue was resected and digested in DMEM/F12 medium containing 0.2% (wt/vol) collagenase type I and 0.1% (wt/vol) BSA at 37 °C with shaking for 1 hr, after dispersion, mature adipocytes were floated on the surface. After three times further gentle dispersion, washes and centrifuge, the pure adipocytes were yielded. The debris containing immune cells, fibroblast and undigested adipose tissue was further separated, filtered through a 250-μm mesh filter and labeled with anti-CD45.1-FITC antibody (#110706, Biolegend, San Diego, CA, USA) for FACS sorting and analysis.

Oil Red O staining

The Oil Red O stock was prepared by 0.5 g Oil Red O (Sigma-aldrich, # 0-0625) in 100 mL isopropanol. Mature adipocytes were rinsed with PBS and then fixed by 4% formaldehyde for 15 min at room temperature. Fresh Oil Red O working solution was prepared by adding 6 mL stock to 4 mL distilled water and then filtered through a 0.22-μm filter. The mature adipocytes were rinsed with PBS for 3 times, and then added Oil Red O working solution for 30 min at 37 °C. The cells were rinse several times carefully with distilled water to remove excess stain and any precipitate that forms and dried to be scanned.

Immunohistochemical staining and evaluation

Tissues were formalin-fixed, paraffin-embedded and then sectioned. After de-paraffinization and rehydration, antigen retrieval was performed with 1 mM EDTA solution (pH 9.0) in a pressurized decloaking chamber for 3 min at 120°C. After peroxidase blocking, the slides were treated with 10% normal goat serum for 1 hr at room temperature followed by the primary antibody incubation. The staining of all slices was blinded, antibodies used: anti-TAZ [1:50 at 37°C overnight (clinical samples)]; [1:100 at 37°C for 1 hr (mouse). Cell Signaling Technology

#4883], anti-Resistin [1:20 at 37°C overnight (clinical samples), Santa Cruz Biotechnology #sc-376336]; [1:200 at 37°C for 1 hr (mouse), PeproTech #500-P182G], Ki67 [1:200 at 37°C 1 hr (clinical samples), ZSGB-BIO (Beijing, China) #ZM-0166]; [1:800 at 4°C overnight (mouse), Cell Signaling Technology #12202]. The slides were then incubated in signal enhancer for 1 hr followed by horseradish peroxidase (HRP)-conjugated secondary antibodies (ZSGB-BIO) for 1 hr at 37°C. After rinsed, the slices were incubated in 3,3'-diaminobenzidine substrate (ZSGB-BIO) and counterstained with hematoxylin. For tissue immunofluorescence staining, the slices were incubated with TAZ (1:800 at 4°C overnight) and Perilipin-1 (1:800 at 4°C overnight). After rinsed, the slices were incubated with DAPI (Invitrogen).

Immunohistochemistry staining was evaluated blindly. For TAZ and Resistin immunostaining in adipose tissue, the scoring criteria is based on staining intensity (SI) and percentage of positive cells (PP). The staining intensity (SI) was determined as 0, negative; 1, weak positive; 2, moderate positive; and 3, strong positive and the percentage of positive cells (PP) was defined as 0, positive in <1%; 1, positive in 1%-25%; 2, positive in 25%-50%; 3, positive in 51%-75%; and 4, positive in >75% positive cells. IHC score = SI × PP. IHC score 0-2 was considered as low expression and 3-12 was considered as high expression of TAZ in adipocytes.

For Ki67 staining analysis, periadipocyte areas were selected as 1 mm radially inward in TNBC samples or 500 μm radially inward in mice tumors along the cancer-adipocyte interface, periphery (no adipocyte) areas were selected as 1 mm radially inward in TNBC samples or 500 μm radially inward in mice tumors from the edge of tumor without adipocytes, interior areas were selected as in tumor interior and 1 mm or 500 μm distant from periadipocyte areas and periphery (no adipocyte) areas in TNBC or mice tumors. 5-10 20-fold fields were randomly selected to quantify the proportion of Ki67+ cells according to the size of the area, the average of all fields in an area was shown in the results.

EdU incorporation assay

The EdU incorporation assay was performed using a Cell-Light EdU Apollo 488 In Vitro Imaging Kit (Ribobio, Guangzhou, China) as previously described(7). Briefly, treated 4T1 and E0771 cells were cultured with 20 μM EdU for 2 hr and then fixed with 4% paraformaldehyde for 30 min at room temperature. Cells were then washed with 2 mg/mL glycine for 5 min followed by washed twice with PBS contained 0.5% Triton X-100. 100 μL Apollo 567 stain reaction buffer was added into the cells and incubated for 30 min in dark. The cells were then washed three times with PBS contained 0.5% Triton X-100 and stained with 100 μL Hoechst 33342 (5 mg/mL) for 30 min at room temperature. EdU-labeled cells were counted in 8 randomly selected fields under a fluorescent microscope.

Chromatin Immunoprecipitation

Totally differentiated 3T3-L1 adipocytes in 15 cm dish were crosslinked with 1% formaldehyde for 15 min at room temperature. Cells were rinsed with ice-cold PBS and lysed in cell lysis buffer containing 5 mM Pipes-KOH (pH 8.0), 85 mM KCl, 0.5 % NP-40 (vol/vol), supplemented with protease inhibitor cocktail (Roche) for 30 min, the supernatant was discarded and nuclei were resuspended in nuclei lysis buffer containing 50 mM Tris-Cl (pH 8.0), 5 mM EDTA, 1% SDS (wt/vol) and cocktail. Keeping the suspension on ice and sonicating chromatin to an average

length of about 500 bp. After centrifugation, a fraction was taken for input, the rest was diluted 10-fold in ChIP dilution buffer containing 20 mM Tris-Cl (pH 8.0), 150 mM NaCl, 2 mM EDTA, 1% Triton X-100 and cocktail. The suspension was pre-cleared with salmon sperm DNA coated protein A agarose beads and then subjected to immunoprecipitations overnight at 4°C (Immunoprecipitation was performed by using specific antibody (anti-PPARγ (#ab45036, abcam), anti-TAZ (#4883, Cell Signaling Technology) /IgG-coated Dynabeads Protein A (#10001D, Invitrogen)). Magnetic beads were washed with low salt wash buffer containing 20 mM Tris-HCl (pH 8.0), 2 mM EDTA, 150 mM NaCl, 0.1 % SDS, 1 % Triton X-100, high salt wash buffer containing 20 mM Tris-HCl (pH 8.0), 2 mM EDTA, 500 mM NaCl, 0.1 % SDS, 1% Triton X-100, LiCl wash buffer containing 10 mM Tris-HCl (pH 8.0), 1 mM EDTA, 0.25 M LiCl, 1% NP-40, 1% deoxycholate, TE buffer containing 10 mM Tris-HCl (pH 8.0), 0.5 M EDTA and eluted in elution buffer (1% SDS, 0.1 M NaHCO3). The eluents and reserved input were reverse-crosslinked by adding 1 μL 10 mg/mL RNase and 0.3 M NaCl and incubated in a 65°C water bath for 5 hr. The supernatant was then added 2.5 volumes of 100 % and precipitated overnight at -20°C. Pellet DNA and debris were resuspended in 100 μL nucleic acid-free water and supplemented with 2 μL of 0.5 M EDTA, 4 μL 1M Tris (pH 6.5) and 1 μL of 20 mg/mL Proteinase K and incubated for 2 hr at 55°C. The DNA was then purified by using spin columns (QIAGEN) and eluted in 100 μL nucleic acid-free water. RT-qPCR analysis was used to measure the enrichment of specific promoter region. All reactions were performed in triplicates. The ChIP-RT-qPCR primers were shown in ***SI Appendix*, Table S3**.

RNA sequencing (RNA-seq) and analysis

mRNA in mouse MAT was extracted and purified using Dynabeads mRNA purification kit (#61006, Invitrogen), mRNAs of five mice were mixed in each group and then fragmented to construct the cDNA library. The cDNA library was sequenced on an Illumina HiSeq 4000 sequencing platform. Adaptor sequences and low-quality reads in raw data were trimmed by Trimmomatic v0.33. Clean reads were then mapped to the Mus musculus reference genome (GRCm38.77) by using TopHat 2.1.1. Reads counts was used to quantify the expression level of each gene by HTSeq. The differentially expressed genes between two groups (adjust p value < 0.05) were performed by DESeq v1.10.1. Kyoto Encyclopedia of Genes and Genomes (KEGG) pathway enrichment analysis for differentially expressed genes was performed by KOBAS 3.0. Gene Set Enrichment Analysis (GSEA) from significantly differentially expressed genes (fold change > 1 and the adjust p value < 0.05) was performed using GSEA v2.0.14 software (http://www.broadinstitute.org/gsea/index.jsp). Heat-map representation of gene expression was generated using R/Morpheus (https://software.broadinstitute.org/morpheus/). For MAT secretome analysis, significantly differentially expressed genes (adjust p value < 0.05) were subjected to SignalP 4.1 (http://www.cbs.dtu.dk/services/SignalP/) to determine the presence of secretion signal peptide.

Allograft experiments

Fresh E0771 cells were resuspended in PBS at a density of $5*10^6$/mL. 100 μL cell suspension were inoculated into the mammary fat pad of the thoracic (glands # 2) mammary glands in CD/HFD female mice through a 22-G needle. 6 days after injection, the length and width of the

mammary tumors were measured with sliding calipers every day. Tumor volume in cm^3 was calculated by the formula: Volume = $(width)^2 \times length/2$. Mice were sacrificed at day 13 when the length of the tumor was less than 1 cm and tumors were resected. Tumor weight was then determined and fixed in 4% paraformaldehyde solution for immunohistochemistry. For Resistin antibody therapy assay, the neutralizing antibody was purified from Resistin peptide (EAIDKKIKQDF-BSA) immunized New Zealand White rabbits by antigen affinity purification. After tumor injection, the antibody in PBS was injected into the mammary fat pad on days 2, 4, 8 and 12 at 0.75 mg/kg body weight. The control group was injected the same volume and amount of rabbit IgG in PBS.

In vivo limiting dilution assay

E0771 cells were cultured with control or TAZ knockdown-Adipo-CM for 8 weeks, and were then prepared in RPMI 1640 medium: Matrigel (#354230, Corning, NY, USA): control or TAZ knockdown-mammosphere formation medium = 2:1:1 buffer and injected into mammary fat pads of 12 week-CD- or HFD-fed-TAZ WT or AKO mice using a cell doses of 5×10^5, 3×10^5, 1×10^5, 5×10^4, 1×10^4 and 1×10^3 /depot, 8 depots per sub-group, the growth of tumor was valuated after a 4-week period, cell stem cell frequency was determined. The estimated CSC frequency was calculated using the limiting dilution software package on the website of Walter and Eliza Hall Institute of Medical Research (http://bioinf.wehi.edu.au/software/elda/index.html).

Statistics

Group size was based on previous experience. No statistical method was used to predetermine sample size. Unless otherwise noted, the results are representative of at least three independent repeats. Data shown in column graphs represent mean ± standard error of the mean (SEM), as indicated in the figure legends, and individual data points are plotted. Statistical analysis was performed with GraphPad Prism 6 (GraphPad Software, San Diego, CA, USA) and SPSS 22.0 (SPSS Inc. Chicago, IL, USA). Details of statistical testing can be found in the figure legends and in the source data file. Unpaired Student’s two-tailed *t* test, one-way ANOVA or two-way ANOVA was used to determine significance. Correlation was studied by Pearson’s Correlation test as indicated. Differences in Kaplan-Meier plots were analyzed by log-rank test. In all assays, $p < 0.05$ was considered statistically significant and was annotated throughout as $^{*}p < 0.05$, $^{**}p < 0.01$, $^{***}p < 0.001$.

***SI Appendix*, Figure Legends**

Fig. S1

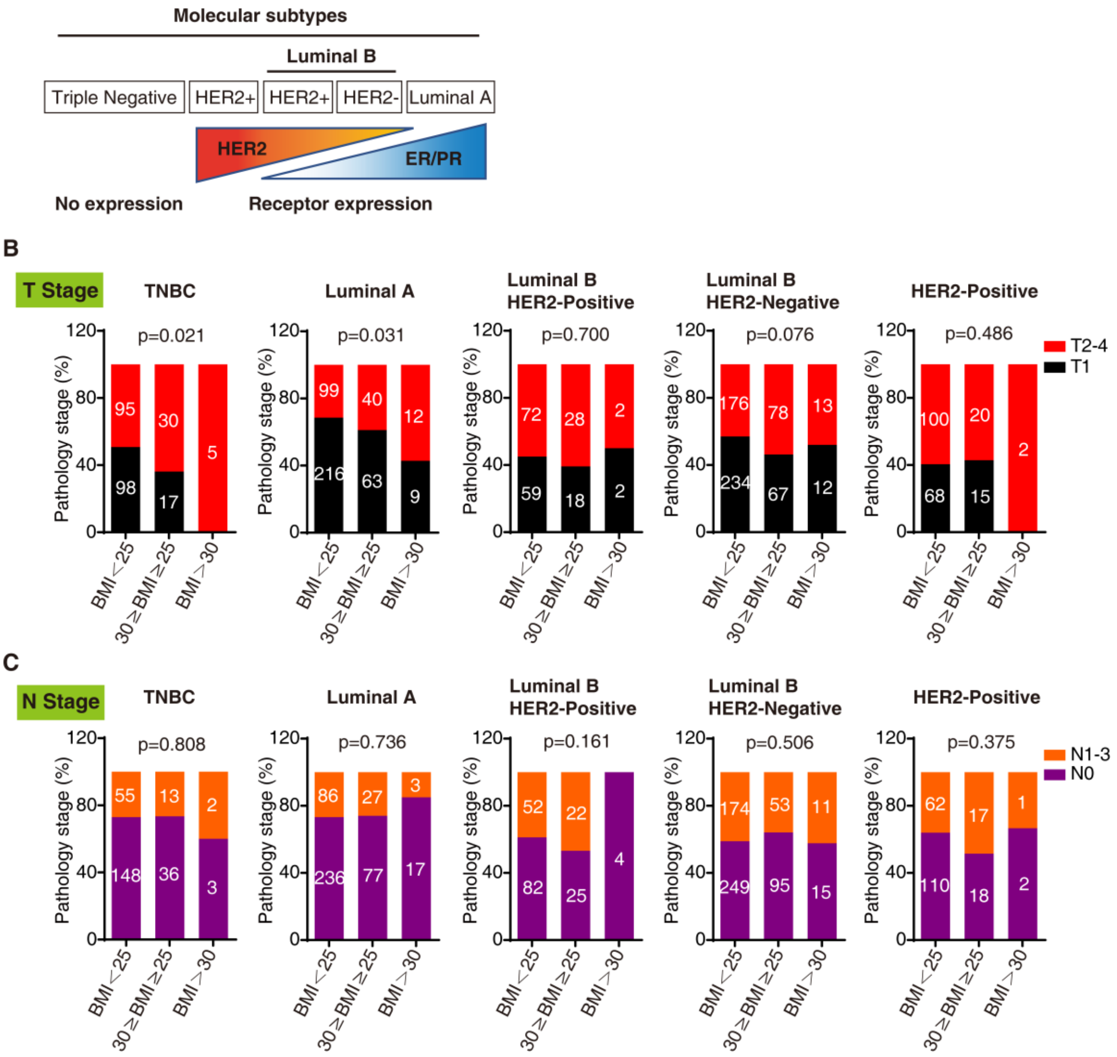

Fig. S1. The correlation between obesity and breast cancer subtypes. (***A***) Molecular subtypes of breast cancer. Breast cancer was divided into five subtypes, Luminal A, Luminal B/HER2-positive, Luminal B/HER2-negative, HER2-negative and TNBC, based on the expression of ER, PR and HER2. (***B***) The distribution of breast cancer by BMI according to T stage. (***C***)The distribution of breast cancer by BMI according to N stage. Data were analyzed using Pearson's Correlation.

Fig. S2

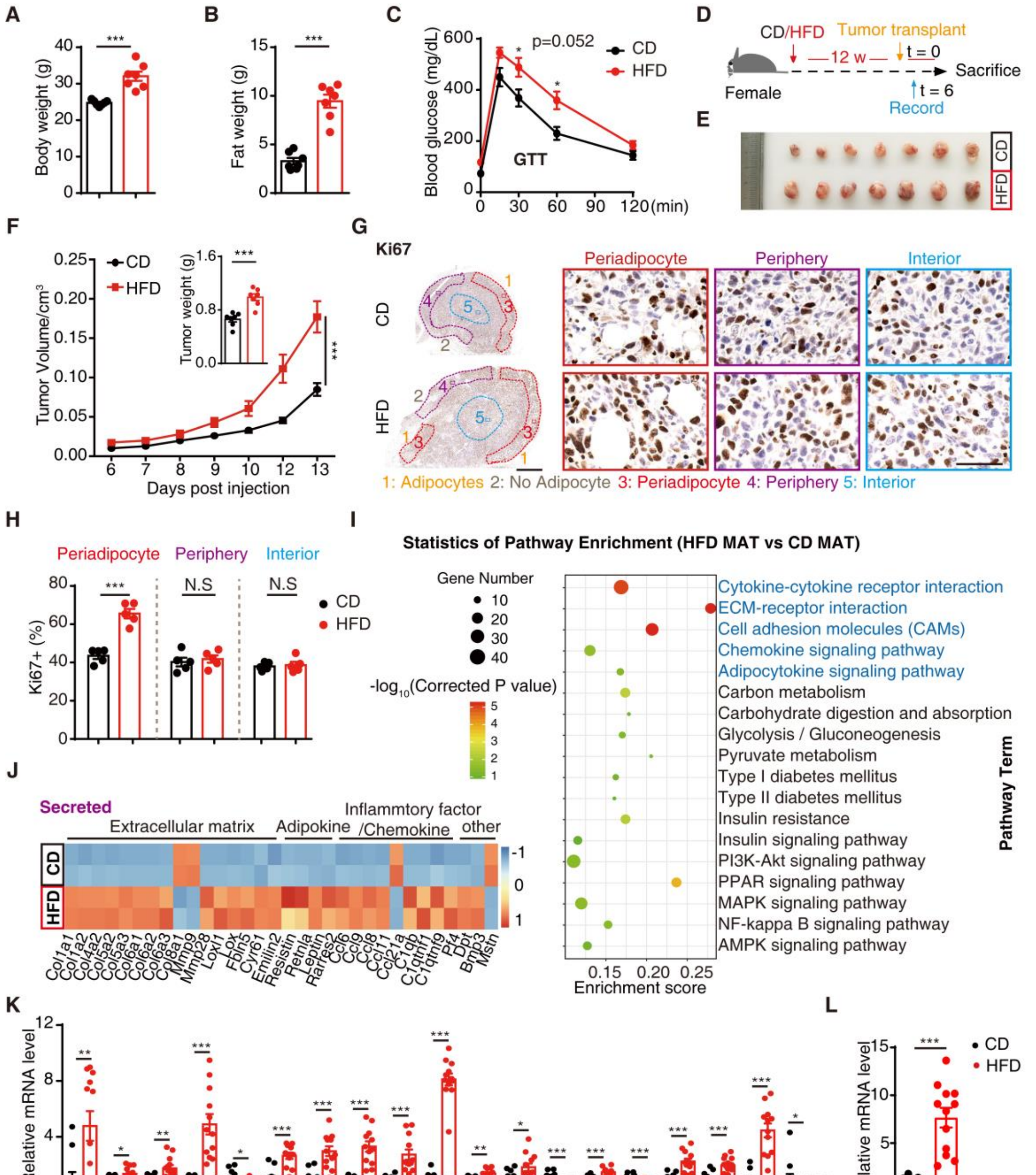

Fig. S2. HFD-induced obesity promotes breast tumor growth and variation of MAT gene profile. (***A***) Female C57BL/6J mice body weights were measured after CD or HFD for 12 weeks. (***B***) Female C57BL/6J mice fat weights were measured by MRI after CD or HFD for 12 weeks. (***C***) GTT assays of female mice fed with a CD or HFD for 12 weeks. n = 7 in each group. (***D***) Trial schematic for diet-induced obesity and breast tumor transplant assay: mice were fed with CD or HFD for 12 weeks and followed by orthotopic injection of E0771 cells. (***E***) Representative image of breast tumors from CD and HFD mice. (***F***) Tumor volumes in CD and HFD mice were measured since palpable at indicated times. Tumor progression are presented. n = 7 per group. After euthanized at day 13, breast tumors from CD and HFD mice were resected, tumor weight was measured. (***G*** and ***H***) Immunohistochemistry staining of Ki67 was performed on tumor

slices, representative images of periadipocyte areas, periphery areas and interior areas were shown (***G***). Scale bar, 1 mm (left) and 50 μm (right). The proportion of Ki67+ cells in each area was quantified from CD and HFD group (***H***). n = 5 per group. (***I***) KEGG pathway analysis of differentially expressed genes identified by RNA-seq in MAT from CD and HFD mice. (***J***) Heatmap of differentially expressed genes that encode secreted proteins in CD- and HFD-MAT. (***K***) RT-qPCR analysis of differentially expressed genes that encode secreted proteins in CD- and HFD-MAT after 12 weeks feeding. (***L***) RT-qPCR analysis of TAZ in CD- and HFD-MAT after 12 weeks feeding. n = 12 in each group. Data shown are mean ± SEM. Data were analyzed using Student's *t* test (***A***, ***B***, ***F***-tumor-weight, ***H***, ***K***, ***L***) and two-way ANOVA (***C***, ***F***-tumor-volume). N.S: no significance, *p < 0.05, **p < 0.01, ***p < 0.001.

Fig. S3

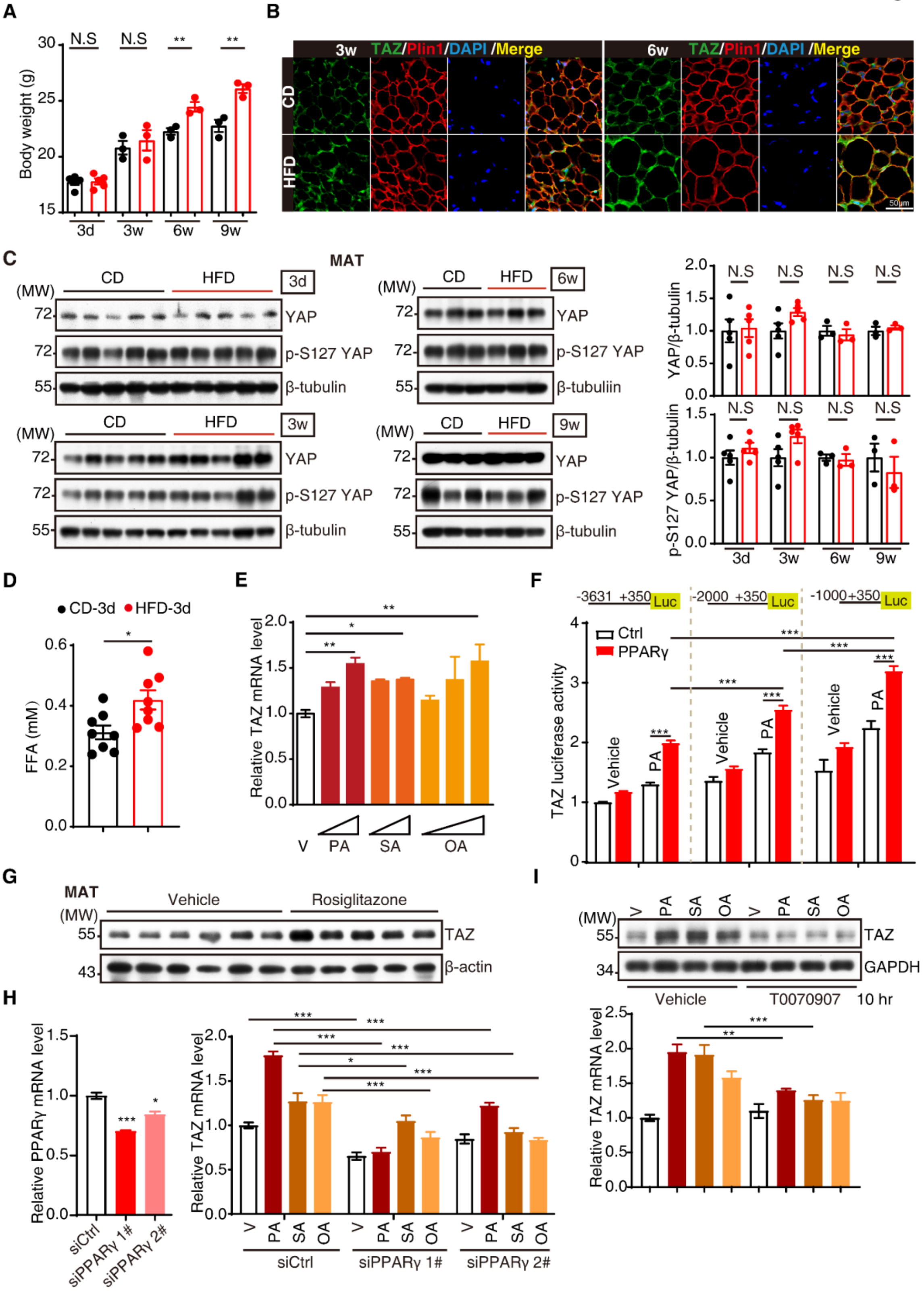

Fig. S3. FFA/ PPARγ axis promotes TAZ expression in adipocytes. (***A***) Female C57BL/6J mice body weight were measured after fed with CD or HFD for indicated times. (***B***) Immunofluorescence staining of TAZ and perilipin1 in MAT from mice fed with CD or HFD for 3 weeks and 6 weeks. (***C***) Western blot analysis of YAP and p-S127 YAP levels in MAT from mice

fed with a CD or HFD for indicated times. Quantification of YAP and p-S127 YAP levels by normalizing to β-tubulin were shown. (***D***) Serum fatty acids levels were measured from mice fed with a CD or HFD for three days. n = 8 per group. (***E***) 3T3-L1 adipocytes were treated with free fatty acids (PA, palmitic acid, 100 μM, 200 μM; SA, stearic acid, 100 μM, 200 μM; OA, oleic acid, 100 μM, 200 μM, 400 μM) or vehicle for 12 hr and then harvested for RT-qPCR analysis. (***F***) 293T cells were co-transfected with truncated TAZ luciferase reporter plasmid and PPARγ, 12 hr after transfection, cells were treated with 400 μM palmitic acid or vehicle for another 12 hr. Cells were then lysed and the reporter value was measured. (***G***) Rosiglitazone (RSG, 1mg/kg body weight, 3 times) was injected into the thoracic (glands # 2) mammary fat pad of 8-week old female C57 BL/6J mice. After treatment, mice were sacrificed and MAT was resected for Western blotting. The protein level of TAZ was quantified. (***H***) 3T3-L1 adipocytes were transfected with siRNA against PPARγ, 60 hr later, cells were treated with 400 μM free fatty acids for another 12 hr. The cells were then harvested for RT-qPCR analysis. The knockdown efficiency was shown (left). (***I***) 3T3-L1 adipocytes were treated with 20 μM T0070907 together with 400 μM free fatty acids or vehicle for 10 hr, cells were then harvested for Western blot analysis (up) and QT-qPCR analysis (down). Data shown are mean ± SEM. Data were analyzed using Student's *t* test (***A***, ***C*** and ***D***), one-way ANOVA (***E*** and ***H***, left) and two-way ANOVA (***F***, ***H***, right and ***I***). N.S: no significance, *p < 0.05, **p < 0.01, ***p < 0.001.

Fig. S4

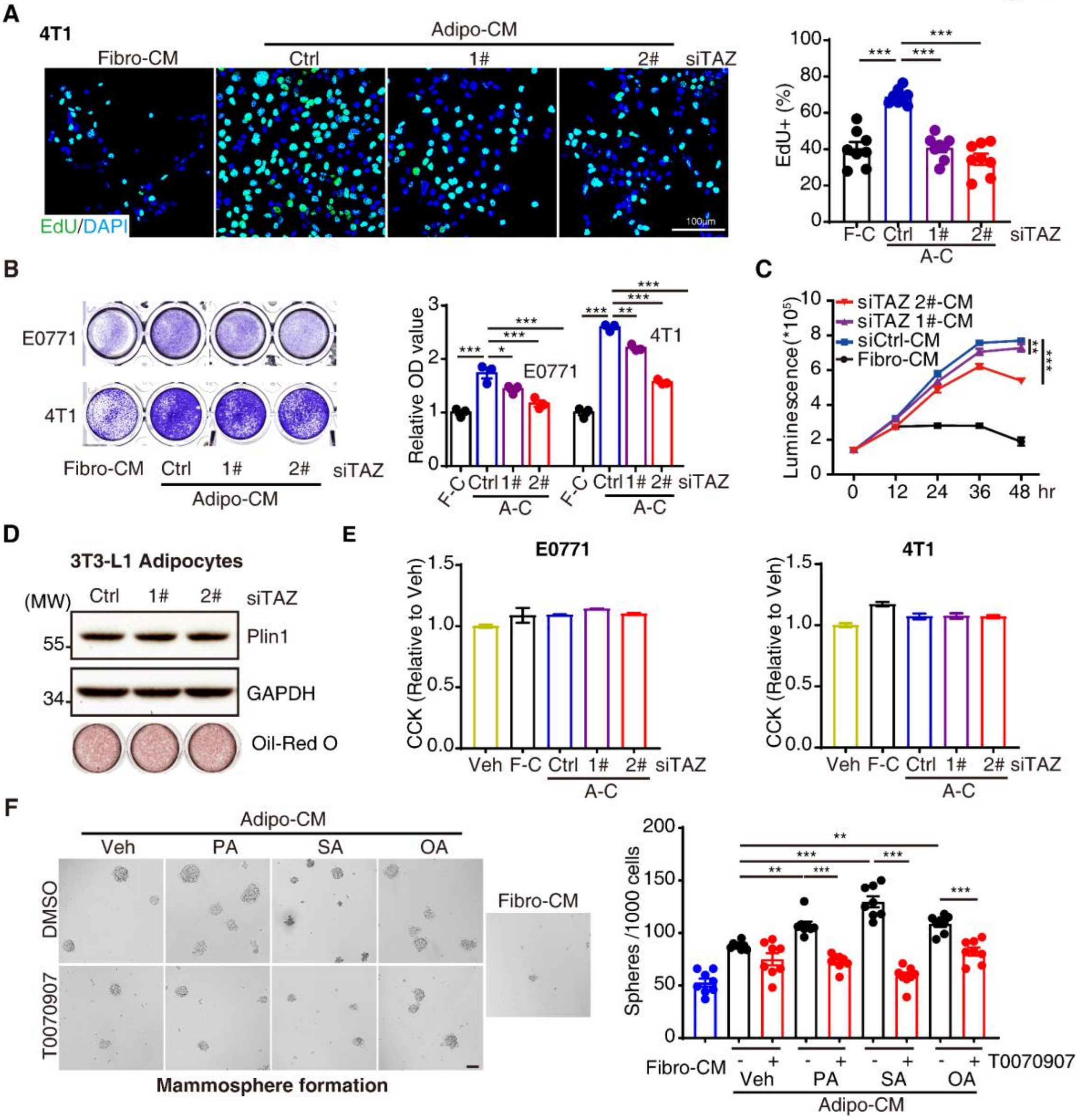

Fig. S4. Adipocyte TAZ promotes breast cancer proliferation. (***A***) 4T1 cells were cultured in control or TAZ knockdown-Adipo-CM for 72 hr and subjected to EdU incorporation assays. The new generation cells were detected *via* EdU (green). DAPI stained nuclei in blue. Merged view of EdU (green) and DAPI (blue) showing the overlap (up), the quantification for EdU staining was shown (down). (***B***) E0771 and 4T1 cells were cultured with control or TAZ knockdown-Adipo-CM plus 100 ng/mL Resistin or vehicle for 8 weeks, and were then plated 5x10^4 cells per well into a 24-well plate with duplicates for another 72 hr-culture, the cells were fixed and stained with crystal violet, representative image (left) and quantification of the OD value at 570 nm (right) was shown. (***C***) E0771 cells were seeded into 96-well plate in control or TAZ knockdown-Adipo-CM or Fibro-CM, every 12 hr since seeding, cells were lysed, exposed to the ATP substrate solution and luminescent signal was measured on a luminescent counter. (***D***) 3T3-L1 adipocytes were incubated with siRNA targeting TAZ, 72 hr after transfection, cells were harvested for Western blot analysis (up) or stained with Oil Red O solution, representative image was shown (down). (***E***) CCK assay of E0771 and 4T1 cells cultured in control or TAZ knockdown-Adipo-CM for 12hr. Veh for Vehicle (DMEM Medium plus 1% BSA), F-C for Fibro-

CM, A-C for Adipo-CM. (***F***) 3T3-L1 adipocytes were treated with free fatty acids (PA, palmitic acid, 400 μM; SA, stearic acid, 400 μM; OA, oleic acid, 400 μM) or vehicle for 12 hr and then washed and cultured with serum-free medium for another 12 hr. Fresh supernatant was harvested and mixed with 15% normal mammosphere formation medium and 0.5% methylcellulose. 1000 E0771 cells were suspension-cultured in the indicated medium for 7 days, the representative images were obtained by microscopy (left), scale bar, 100 μm. Mammosphere numbers were counted and analyzed (right). Data shown are mean ± SEM. Data were analyzed using one-way ANOVA (***A*** and ***B***) and two-way ANOVA (***C*** and ***F***). $^{*}p < 0.05$, $^{**}p < 0.01$, $^{***}p < 0.001$.

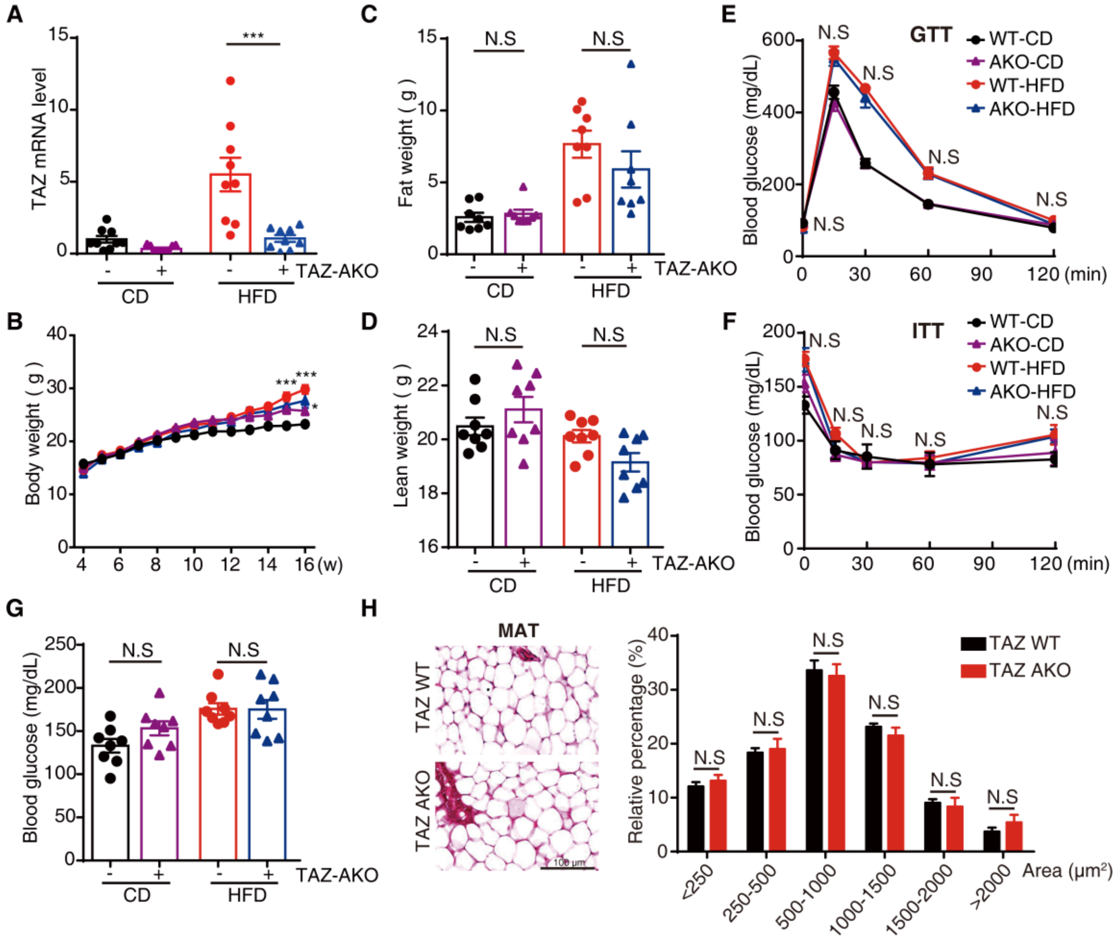

Fig. S5. Adipocytic TAZ knockout does not affect glucose metabolism and adipocyte size in female mice. (***A***) MATs from TAZ WT and AKO tumor-bearing mice in CD and HFD groups were lysed and analyzed the expression of TAZ by RT-qPCR. n = 9 in each group. (***B***) Body weight of female TAZ WT and AKO mice fed with CD or HFD. n = 9 in each group. (***C*** and ***D***) Fat weights (***C***) and body lean weights (**D**) of TAZ WT and TAZ AKO mice fed with CD or HFD for 12 weeks were measured by MRI. n = 8 in each group. (***E***) GTT assays of TAZ WT and TAZ AKO mice fed with CD or HFD for 12 weeks. n = 8 in each group. (***F***) ITT assays of TAZ WT and TAZ AKO mice fed with CD or HFD for 12 weeks. n = 8 in each group. (***G***) Quantification of serum glucose concentrations of TAZ WT and TAZ AKO mice fed with CD or HFD. n = 8 in each group. (***H***) Hematoxylin and eosin staining of MAT from TAZ WT and AKO mice (left), the area of the adipocyte was calculated by Image J software, and the relative percentage was shown (right). Data shown are mean ± SEM. Data were analyzed using two-way ANOVA. N.S: no significance, *p < 0.05, ***p < 0.001.

Fig. S6

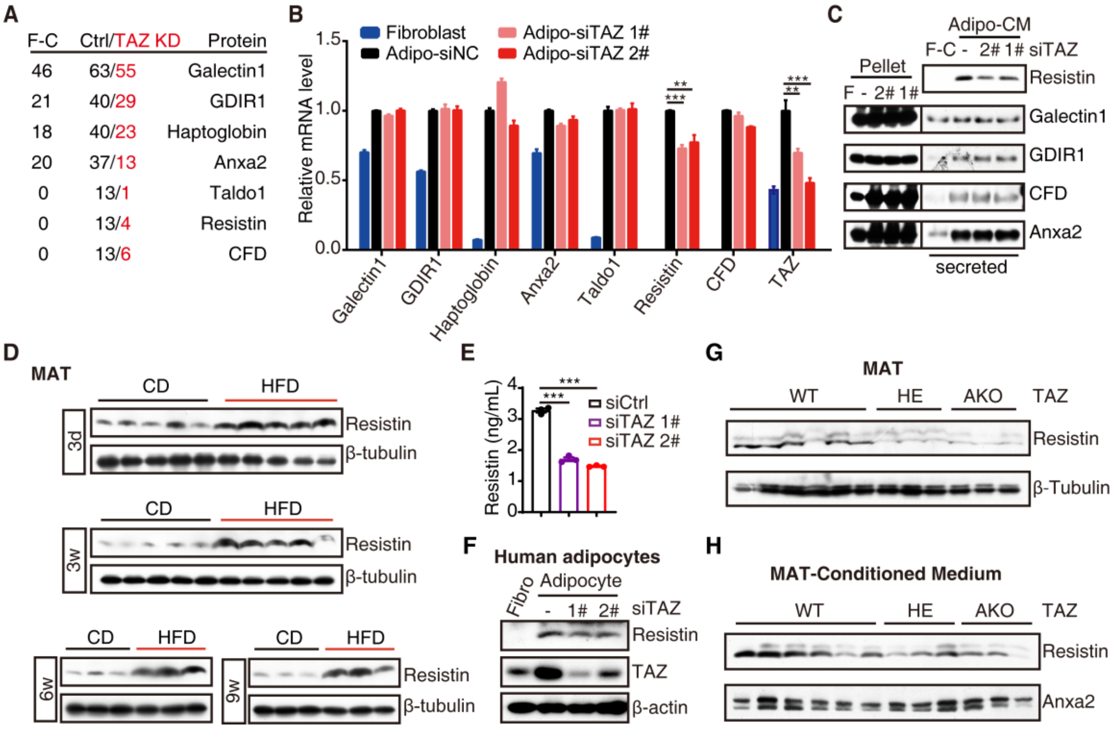

Fig. S6. Mass spectrometry identifies Resistin as a target of TAZ in adipocytes. (***A***) Mass spectrometry analysis of control or TAZ knockdown-Adipo-CM or Fibro-CM. The list shows the differentially secreted protein in the medium. (***B***) 3T3-L1 fibroblasts and 3T3-L1 adipocytes transfected with control or siRNA targeting TAZ were harvested for RT-qPCR analysis of genes screened from mass spectrometry. (***C***) Cell pellets and conditioned medium of 3T3-L1 fibroblasts and 3T3-L1 adipocytes transfected with control siRNA or siRNA targeting TAZ were harvested for Western blot analysis of secreted proteins screened from mass spectrometry. F, fibroblast, F-C, Fibro-CM. (***D***) Western blot analysis of Resistin expression in MAT after fed with CD or HFD for indicated times. (***E***) 3T3-L1 adipocytes were transfected with siRNA targeting TAZ, 72 hr after transfection, cells were washed and cultured with serum-free medium for another 12 hr. Resistin levels in the supernatant was determined by ELISA. (***F***) Human preadipocytes (Fibro) were differentiated into mature adipocytes and then transfected with TAZ siRNA, 72 hr after transfection, cells were harvested for Western blot analysis with indicated antibodies. (***G***) MATs from TAZ WT, HE (heterozygous) and AKO mice were resected and lysed for Western blot analysis with indicated antibodies. (***H***) MATs from TAZ WT, HE (heterozygous) and AKO mice were resected and cultured in serum free medium for 1 hr, secreted proteins in supernatants were precipitated for Western blot analysis. Data shown are mean ± SEM. Data were analyzed using one-way ANOVA. **$p < 0.01$, ***$p < 0.001$.

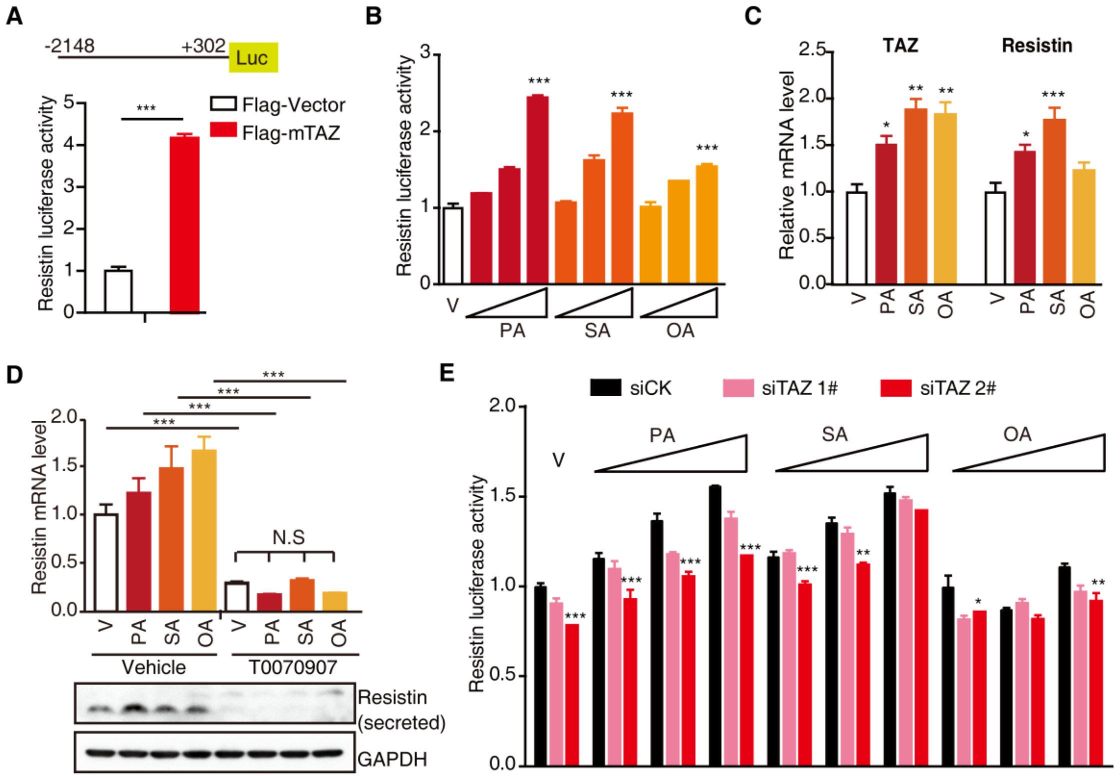

Fig. S7. FFA/ PPARγ/ TAZ axis promotes Resistin expression in adipocytes. (***A***) 293T cells were co-transfected with Resistin luciferase reporter plasmid and TAZ, 16 hr after transfection, cells were lysed and the reporter value was measured. (***B***) 293T cells were transfected with Resistin luciferase reporter plasmid, 12 hr after transfection, cells were treated with indicated free fatty acids (100 μM, 200 μM, 400 μM) or vehicle for another 12 hr. Cells were then lysed and the reporter values were measured. (***C***) 3T3-L1 adipocytes were treated with free fatty acids (PA, palmitic acid, 400 μM; SA, stearic acid, 400 μM; OA, oleic acid, 400 μM) or vehicle for 16 hr and then harvested for RT-qPCR analysis. (***D***) 3T3-L1 adipocytes were pretreated with 20 μM T0070907 for 6 hr, and then treated with 400 μM indicated free fatty acid in serum free medium for 12 hr. The supernatants were then collected and precipitated for Western blot (down), the cells were harvested for RT-qPCR (up) and Western blot (down) analysis. (***E***) 293T cells were transfected with control siRNA or siRNA targeting TAZ for 48 hr followed by transfection with Resistin luciferase reporter plasmid, 12 hr after transfection, cells were treated with indicated free fatty acids (100 μM, 200 μM, 400 μM) or vehicle for another 12 hr. Cells were then lysed and the reporter value was measured. Data shown are mean ± SEM. Data were analyzed using Student's *t* test (***A***), one-way ANOVA (***B*** and ***C***) and two-way ANOVA (***D*** and **E**). N.S: no significance, *p < 0.05, **p < 0.01, ***p < 0.001.

Fig. S8

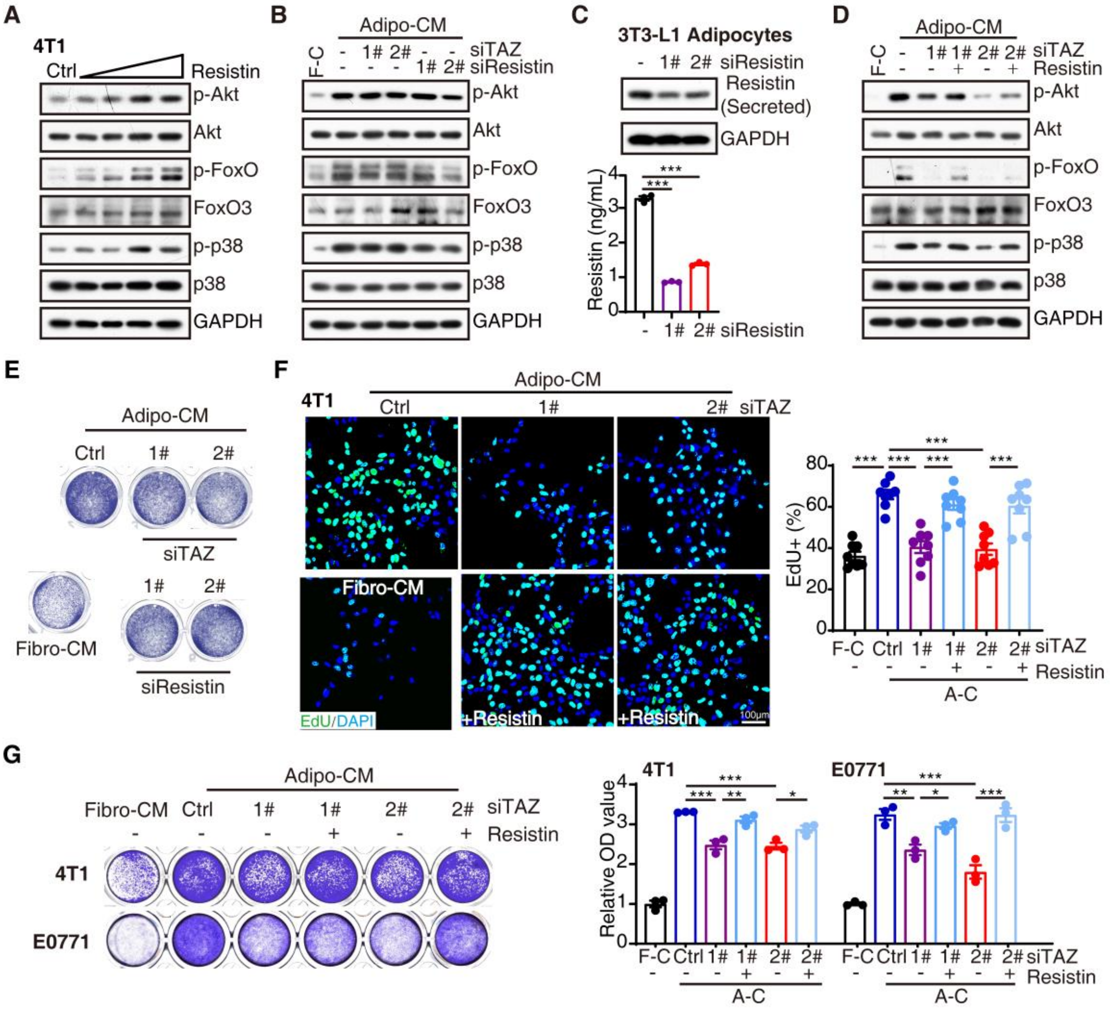

Fig. S8. Resistin promotes breast cancer proliferation. (***A***) 4T1 cells were washed and incubated with vehicle or 10, 100, 500, 1000 ng/mL Resistin for 30 min and then harvested for Western blot analysis. (***B***) 4T1 cells were incubated with control or TAZ/Resistin knockdown-Adipo-CM for 30 min and then harvested for Western blot analysis. (***C***) 4T1 cells were incubated with control or TAZ knockdown-Adipo-CM supplemented with 1 μg/ml Resistin or vehicle for 30 min and then harvested for Western blotanalysis. (***D***) 3T3-L1 adipocytes were transfected with siRNA targeting Resistin, 72 hr after transfection, cells were washed and cultured with serum-free medium for another 12 hr. The cells were collected and supernatant was precipitated for Western blot analysis, Resistin levels in the supernatant was determined by ELISA. (***E***) 4T1 cells were cultured in control or TAZ/Resistin knockdown adipo-CM. 60 hr after coculture, 4T1 cells were fixed and stained with crystal violet, representative image was shown. (***F*** and ***G***) 4T1 cells were cultured in control or TAZ knockdown-Adipo-CM plus 100 ng/mL Resistin or vehicle and then subjected to EdU incorporation assays. The new generation cells were detected *via* EdU (green). DAPI stained nuclei in blue. Merged view of EdU (green) and DAPI (blue) showing the overlap (***F***), the quantification for EdU staining was shown (***G***). (***H***) E0771 and 4T1 cells were cultured with control or TAZ knockdown-Adipo-CM plus 100 ng/mL Resistin or vehicle for 14 days, and were then plated $5x10^4$ cells per well into a 24-well plate with duplicates for another 72 hr-culture, the cells were fixed and stained with crystal violet, representative image

(left) and quantification of the OD value at 570 nm (right) was shown. Data shown are mean ± SEM. Data were analyzed using one-way ANOVA. *$p < 0.05$, **$p < 0.01$, ***$p < 0.001$.

Fig. S9

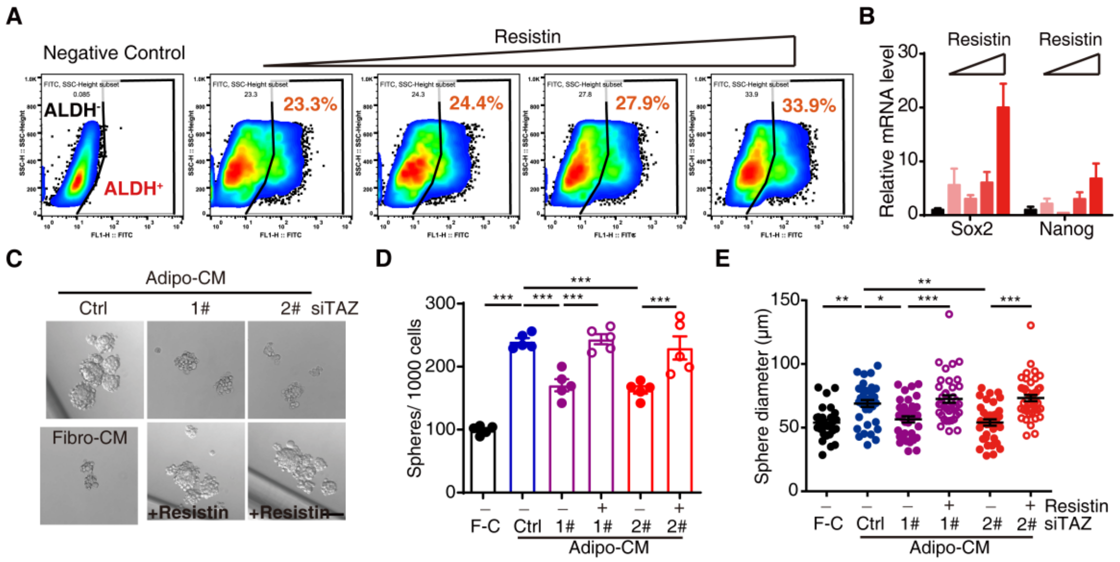

Fig. S9. TAZ/ Resistin axis promotes breast cancer cell stemness. (***A*** and **B**) E0771 cells were cultured with 50, 100, 500,1000 ng/ml Resistin or vehicle for 1 week, and then incubated with fluorochrome-conjugated ALDH1 antibody and analyzed by flow cytometry (***A***) or harvested for RT-qPCR analysis (***B***). (***C-E***) 1000 E0771 cells were suspension-cultured in mammosphere formation medium containing control or TAZ knockdown-Adipo-CM supplemented with 1 μg/mL Resistin or vehicle for 10 days, the representative images were obtained by microscopy (***C***), scale bar, 100 μm. Mammosphere number was counted and shown (***D***). The diameter of mammosphere was measured by Image J software and shown (***E***). Data were analyzed using one-way ANOVA. *$p < 0.05$, **$p < 0.01$, ***$p < 0.001$.

Fig. S10

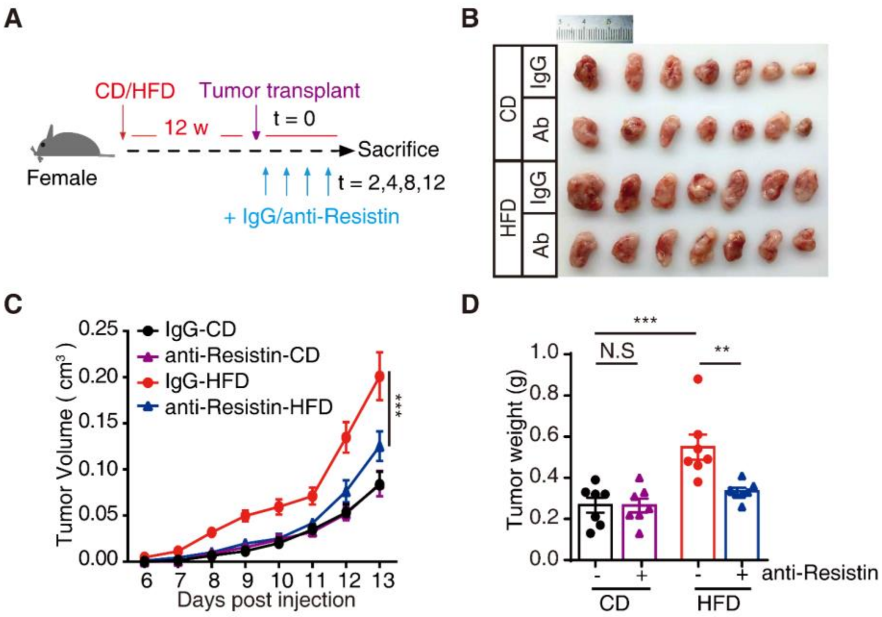

Fig. S10. A neutralizing antibody to Resistin mitigates HFD-induced breast cancer growth. (***A***) Trial schematic for mice feeding and breast tumor transplant assay and strategy for Resistin neutralizing antibody therapy. Briefly, female mice were enrolled on a CD or HFD for 12 weeks, at day 0, E0771 cells were orthotopically injected into fat pads, the Resistin antibody or control IgG was injected the into the MAT around the tumor at day 2, 4, 8 and 12 at 0.75 mg/kg body weight. The tumor growth was monitored. (***B***) Representative image of breast tumors from IgG and neutralizing antibody therapy groups of CD and HFD mice. Ab for antibody. (***C***) Tumor volumes in IgG and neutralizing antibody therapy groups of CD or HFD mice were measured since palpable at indicated times. Tumor progression are presented. n = 7 per group. (***D***) All the mice were euthanized at day 13, breast tumor was resected, tumor weight was measured. Data shown are mean ± SEM. Data were analyzed using two-way ANOVA. $^{**}p < 0.01$, $^{***}p < 0.001$.

Fig. S11

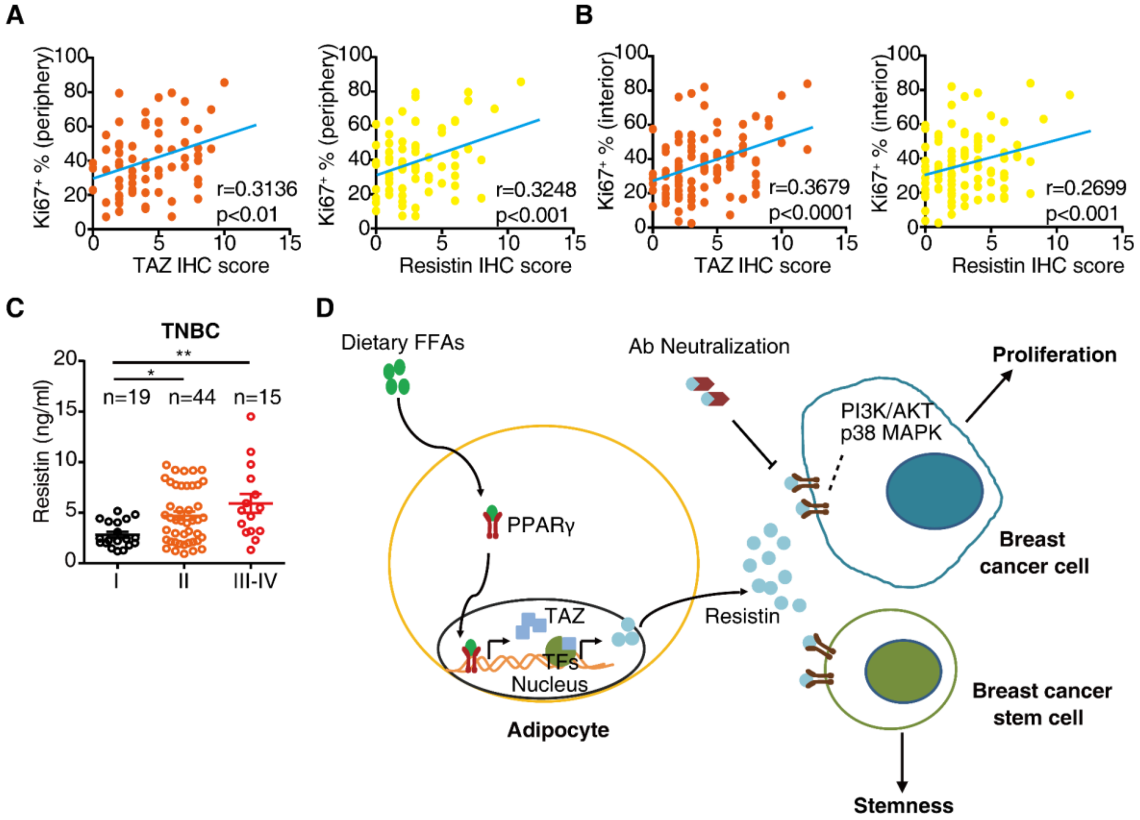

Fig. S11. Clinical relevance of TAZ and Resistin in TNBC. (***A*** and ***B***) Scatterplots of periphery (***A***, n=70) or interior (***B***, n=96) Ki67+ proportion related to TAZ or Resistin IHC scores in patient samples (n = 112). (***C***) ELISA analysis of serum Resistin levels of 78 TNBC patients from different clinical stages. (***D***) Schematic representation of the proposed roles of adipocyte TAZ/Resistin facilitating breast cancer cell proliferation and stemness. FFA/PPARγ transcriptionally upregulates the expression of TAZ in adipocytes, the elevated TAZ promotes the expression and secretion of Resistin and therefore facilitates breast cancer cell proliferation and stemness maintenance. Data shown are mean ± SEM. Data were analyzed using one-way ANOVA (***C***) and Pearson's Correlation (***A*** and ***B***). $^{*}p < 0.05$, $^{**}p < 0.01$

Table S1. GSEA top three gene sets

	Name	Probe	Rank in Gene list	Rank Metric Score	Running ES	Core Enrichment	Related to Obesity?
**Gene set 1 (REN_ALVEOLAR_RHABDOMYOSARCOMA_DN)**	row_0	Emp1	118	2.897326	-0.02545	Yes	N/A
	row_1	Cdr2	138	2.731356	0.000276	Yes	N/A
	row_2	Anxa2	171	2.495995	0.015898	Yes	**Yes**
	row_3	Mrc2	174	2.480284	0.047485	Yes	N/A
	row_4	Srpx2	240	2.257568	0.042177	Yes	N/A
	row_5	Col6a2	250	2.204032	0.066353	Yes	**Yes**
	row_6	Lox	252	2.194213	0.094711	Yes	**Yes**
	row_7	Nnmt	259	2.185622	0.120262	Yes	**Yes**
	row_8	Creb3l1	264	2.166852	0.146643	Yes	N/A
	row_9	Ptrf	268	2.157447	0.17344	Yes	**Yes**
	row_10	Thbs1	270	2.154953	0.201282	Yes	**Yes**
	row_11	Fbln5	279	2.139805	0.22515	Yes	N/A
	row_12	Thbs2	281	2.124394	0.25259	Yes	N/A
	row_13	Col6a1	287	2.10131	0.277569	Yes	**Yes**
	row_14	Anxa5	299	2.057065	0.298731	Yes	N/A
	row_15	Snx7	306	2.042931	0.322402	Yes	N/A
	row_16	Anxa1	319	2.008801	0.342389	Yes	**Yes**
	row_17	Cav1	342	1.949032	0.356198	Yes	**Yes**
	row_18	Timp1	354	1.909258	0.375413	Yes	**Yes**
	row_19	Pea15a	383	1.863339	0.384859	Yes	**Yes**
	row_20	Itgbl1	393	1.831439	0.404127	Yes	N/A
	row_21	Col5a1	395	1.827299	0.427654	Yes	**Yes**
	row_22	Tfpi2	399	1.815819	0.449951	Yes	**Yes**
	row_23	Fstl3	416	1.776919	0.464728	Yes	**Yes**
	row_24	Cyr61	430	1.755762	0.480844	Yes	N/A
	row_25	Gsn	474	1.651739	0.479417	Yes	**Yes**
	row_26	Col5a2	487	1.632231	0.494444	Yes	**Yes**
	row_27	Vcan	488	1.630573	0.515919	Yes	N/A
	row_28	Dram1	492	1.625296	0.535707	Yes	N/A
	row_29	Reep5	497	1.618634	0.554868	Yes	N/A
	row_30	Fstl1	502	1.605274	0.573854	Yes	**Yes**
	row_31	S100a10	520	1.578652	0.58548	Yes	N/A
	row_32	Col1a1	522	1.569777	0.605615	Yes	**Yes**
	row_33	Ppic	555	1.525352	0.608454	Yes	N/A
	row_34	Cd248	559	1.517787	0.626826	Yes	N/A
	row_35	S100a6	562	1.51498	0.6457	Yes	N/A
	row_36	Adamts2	575	1.50715	0.65908	Yes	**Yes**
	row_37	Dusp14	744	-1.89452	0.593465	No	**Yes**
	row_38	Slc25a1	777	-1.92881	0.601617	No	N/A

	Name	Probe	Rank in Gene list	Rank Metric Score	Running ES	Core Enrichment	Related to Obesity?
**Gene set 2 (KOIN2-UMA_TARGETS_OF_SMAD2_OR_SMAD3)**	row_0	Serpine1	23	3.916416	0.034228	Yes	N/A
	row_1	Olr1	110	2.952538	0.022978	Yes	**Yes**
	row_2	Arhgef37	117	2.904016	0.054324	Yes	N/A
	row_3	Emp1	118	2.897326	0.088828	Yes	N/A
	row_4	Bcat1	123	2.869725	0.120844	Yes	**Yes**
	row_5	Anxa2	171	2.495995	0.125205	Yes	**Yes**
	row_6	Bcar3	224	2.305496	0.124598	Yes	N/A
	row_7	Pkp2	236	2.271985	0.145718	Yes	N/A
	row_8	Cdkn1a	244	2.236776	0.168578	Yes	**Yes**
	row_9	Pmepa1	257	2.189776	0.18818	Yes	N/A
	row_10	Thbs1	270	2.154953	0.207367	Yes	**Yes**
	row_11	Lgals3	278	2.140143	0.229076	Yes	**Yes**
	row_12	Snx7	306	2.042931	0.238834	Yes	N/A
	row_13	Cav1	342	1.949032	0.243157	Yes	**Yes**
	row_14	Sptbn1	379	1.874645	0.246054	Yes	**Yes**
	row_15	Gpnmb	381	1.869094	0.267773	Yes	**Yes**
	row_16	Itgbl1	393	1.831439	0.283647	Yes	N/A
	row_17	Tfpi2	399	1.815819	0.302573	Yes	**Yes**
	row_18	Vim	404	1.809107	0.321959	Yes	**Yes**
	row_19	Slc7a8	415	1.779723	0.337757	Yes	N/A
	row_20	Cyr61	430	1.755762	0.351111	Yes	N/A
	row_21	Adm	432	1.750875	0.371422	Yes	**Yes**
	row_22	Wwtr1	454	1.698595	0.380318	Yes	N/A
	row_23	Prnp	455	1.696115	0.400516	Yes	N/A
	row_24	Rab38	475	1.651307	0.409928	Yes	N/A
	row_25	Sulf2	490	1.626956	0.421748	Yes	Yes
	row_26	Tubb2a	501	1.607422	0.435494	Yes	N/A
	row_27	Slc37a2	509	1.595893	0.450722	Yes	N/A
	row_28	Serf1	513	1.592652	0.468069	Yes	N/A
	row_29	Sh3pxd2a	517	1.587488	0.485356	Yes	N/A
	row_30	S100a10	520	1.578652	0.503076	Yes	N/A
	row_31	Dhrs3	544	1.538298	0.508983	Yes	N/A
	row_32	Fam83a	565	1.513733	0.516217	Yes	N/A
	row_33	Slc48a1	582	1.497801	0.525419	Yes	N/A
	row_34	Mtfp1	688	-1.75719	0.489681	No	N/A
	row_35	Mpzl2	718	-1.86111	0.496194	No	N/A
	row_36	Dusp14	744	-1.89452	0.505264	No	**Yes**
	row_37	Mmp9	804	-1.96361	0.496808	No	**Yes**
	row_38	Prkab2	836	-2.01438	0.504068	No	N/A
	row_39	Clcf1	945	-2.1887	0.471849	No	N/A
	row_40	B3gnt5	1390	-3.29286	0.271452	No	N/A

	Name	Probe	Rank in Gene list	Rank Metric Score	Running ES	Core Enrichment	Related to Obesity?
Gene set 3 (IGLESIAS_E2F_TARGETS_UP)	row_0	Tuba1a	54	3.503052	0.024284	Yes	N/A
	row_1	Emp1	118	2.897326	0.034521	Yes	N/A
	row_2	Sparc	119	2.896421	0.078579	Yes	**Yes**
	row_3	Col15a1	162	2.542744	0.094701	Yes	**Yes**
	row_4	Lgals1	164	2.532634	0.132688	Yes	**Yes**
	row_5	Anxa2	171	2.495995	0.167433	Yes	**Yes**
	row_6	Col4a1	227	2.29917	0.172868	Yes	**Yes**
	row_7	Serpinf1	249	2.206475	0.195153	Yes	**Yes**
	row_8	Col6a2	250	2.204032	0.228679	Yes	**Yes**
	row_9	Thbs1	270	2.154953	0.251254	Yes	**Yes**
	row_10	S100a11	294	2.080702	0.270551	Yes	N/A
	row_11	Anxa1	319	2.008801	0.288218	Yes	**Yes**
	row_12	Nid1	341	1.952225	0.306636	Yes	N/A
	row_13	Lpl	346	1.925519	0.333777	Yes	**Yes**
	row_14	Nid2	359	1.897671	0.356198	Yes	N/A
	row_15	Col6a3	397	1.824643	0.364082	Yes	**Yes**
	row_16	Col18a1	398	1.822334	0.391801	Yes	**Yes**
	row_17	Tuba1b	434	1.742772	0.399514	Yes	N/A
	row_18	Gsn	474	1.651739	0.403694	Yes	**Yes**
	row_19	Col5a2	487	1.632231	0.422077	Yes	**Yes**
	row_20	Adipoq	494	1.622328	0.443532	Yes	**Yes**
	row_21	Tubb2a	501	1.607422	0.464761	Yes	N/A
	row_22	Hba-a1	505	1.601022	0.487503	Yes	**Yes**
	row_23	S100a10	520	1.578652	0.503997	Yes	N/A
	row_24	Col1a1	522	1.569777	0.527338	Yes	**Yes**
	row_25	Col3a1	527	1.561263	0.548939	Yes	**Yes**
	row_26	Serpinh1	556	1.524585	0.557092	Yes	N/A
	row_27	S100a6	562	1.51498	0.577451	Yes	N/A
	row_28	Fbln2	578	1.500701	0.592223	Yes	**Yes**
	row_29	Cfd	721	-1.86428	0.544318	No	**Yes**
	row_30	Slc25a4	892	-2.1056	0.485047	No	N/A
	row_31	Cxcl13	1429	-3.41918	0.249194	No	N/A

Table S2. Mass spectrometry results

Differentially secreted proteins in the Conditioned Mediums				
		**Fibro-CM**	**Control-Adipo-CM**	**TAZ knockdown-Adipo-CM**
**prot_acc**	**prot_desc**	**prot_matches_sig**	**prot_matches_sig**	**prot_matches_sig**
LEG1_MOUSE	Galectin-1 OS=Mus musculus GN=Lgals1 PE=1 SV=3	46	63	55
PRDX1_MOUSE	Peroxiredoxin-1 OS=Mus musculus GN=Prdx1 PE=1 SV=1	27	51	45
LIPL_MOUSE	Lipoprotein lipase OS=Mus musculus GN=Lpl PE=1 SV=3	11	44	38
GDIR1_MOUSE	Rho GDP-dissociation inhibitor 1 OS=Mus musculus GN=Arhgdia PE=1 SV=3	21	40	29
HPT_MOUSE	Haptoglobin OS=Mus musculus GN=Hp PE=1 SV=1	18	40	23
ANXA2_MOUSE	Annexin A2 OS=Mus musculus GN=Anxa2 PE=1 SV=2	20	37	13
HPT_MUSCR	Haptoglobin OS=Mus caroli GN=Hp PE=2 SV=1	15	36	24
APOD_MOUSE	Apolipoprotein D OS=Mus musculus GN=Apod PE=2 SV=1	26	24	36
CO4B_MOUSE	Complement C4-B OS=Mus musculus GN=C4b PE=1 SV=3	7	23	16
ANXA1_MOUSE	Annexin A1 OS=Mus musculus GN=Anxa1 PE=1 SV=2	13	18	8
CO3_MOUSE	Complement C3 OS=Mus musculus GN=C3 PE=1 SV=3	30	13	17
TALDO_MOUSE	Transaldolase OS=Mus musculus GN=Taldo1 PE=1 SV=2	0	13	1
RETN_MOUSE	Resistin OS=Mus musculus GN=Retn PE=1 SV=1	0	13	4
CFAD_MOUSE	Complement factor D OS=Mus musculus GN=Cfd PE=1 SV=1	0	11	6
FRIL1_MOUSE	Ferritin light chain 1 OS=Mus musculus GN=Ftl1 PE=1 SV=2	7	10	7
CYB5_MOUSE	Cytochrome b5 OS=Mus musculus GN=Cyb5a PE=1 SV=2	2	9	6

HPT_MUSSA	Haptoglobin OS=Mus saxicola GN=Hp PE=2 SV=2	2	8	5
GDIR2_MOUSE	Rho GDP-dissociation inhibitor 2 OS=Mus musculus GN=Arhgdib PE=1 SV=3	5	7	3
TIMP1_MOUSE	Metalloproteinase inhibitor 1 OS=Mus musculus GN=Timp1 PE=1 SV=2	9	7	8
FRIL2_MOUSE	Ferritin light chain 2 OS=Mus musculus GN=Ftl2 PE=2 SV=2	3	6	4
DAG1_MOUSE	Dystroglycan OS=Mus musculus GN=Dag1 PE=1 SV=4	4	5	1
HSP72_MOUSE	Heat shock-related 70 kDa protein 2 OS=Mus musculus GN=Hspa2 PE=1 SV=2	0	4	0
CO6A1_MOUSE	Collagen alpha-1(VI) chain OS=Mus musculus GN=Col6a1 PE=2 SV=1	7	14	9

Table S3. siRNA and ChIP-RT-qPCR primer sequences

siRNA sequences

siRNA (Mouse)	Sequence (5'-3')
**TAZ 1#**	GAUGAAUCCGUCCUCGGUGCC
**TAZ 2#**	CCAUGAGCACAGAUAUGAGAU
**TAZ 3#**	CAGCCGAAUCUCGCAAUGAAU
**Resistin 1#**	GCCCGCUGCUGTAAGCUGCAG
**Resistin 2#**	AGGUCGCUUCCUGAUGUCGGU
**PPARγ 1#**	CGAAGAACCAUCCGAUUGA
**PPARγ 2#**	AAGACCACUCGCAUUCCUUUG
**siRNA (Human)**	**Sequence (5'-3')**
**TAZ 1#**	ACGUUGACUUAGGAACUUU
**TAZ 2#**	AGAGGUACUUCCUCAAUCA

Primer sequences for TAZ promoter ChIP

ChIP primer	Forward primer	Reverse primer
**Primer4 (-699 - -436)**	CCCACTACCCTCGCAAACG	TCCACGGAAGGCTGCTTAA
**Primer3 (-468 - -285)**	TATGGTCACAAGCGTTAAGCAG	GGGTCCAAGGCCCAGAAT
**Primer2 (-306 - -153)**	ACTCATTCTGGGCCTTGGAC	AGCGCCTATCTGCATTCCTT
**Primer1 (-193 - -39)**	CCCAAGTCAGTGGTAAACTCAAA	CTCCTCTTCCTCCTCCTCCC
**Primer5 (-967 - -709)**	AGCAGCCACCCTTTCTTG	ATGTCCCTGGGTCGCTCT

Primer sequences for Resisitn promoter ChIP

ChIP primer	Forward primer	Reverse primer
**Primer1 (-598 - -357)**	GCTCTTGCCTAGACTCCTC	ACACCACCAGACCCTCAC
**Primer2 (-110 - +85)**	CCTCCTCTGGGACCTCTA	GCTATCACTTACCGTGGC
**Primer3 (-295 - -50)**	CCCGCCAAACATAGAAAC	CATTGCCACATCTGACCC
**Primer4 (-375 - -217)**	AGTGAGGGTCTGGTGGTG	CCTGTAGGGCTGGAGATG
**Primer5 (-834 - -544)**	GTCACGCACATGCAATAA	AGGAGGTTCCCAGCCATT

Table S4. RT-qPCR primers sequences

Gene	Forward primer	Reverse primer
**β-actin**	GGCTGTATTCCCCTCCATCG	CCAGTTGGTAACAATGCCATGT
**18S**	CTGCCGTCTGAGTGTATCGC	GCTGGGGCTGAGGAAAGTG
**TAZ**	CATGGCGGAAAAAGATCCTCC	GTCGGTCACGTCATAGGACTG
**Resistin**	AAGAACCTTTCATTTCCCCTCCT	GTCCAGCAATTTAAGCCAATGTT
**Galectin1**	AACCTGGGGAATGTCTCAAAGT	GGTGATGCACACCTCTGTGA
**GDIR1**	AAGGACGATGAAAGCCTCCG	GGTCAGTCGAGTCACAATGACA
**Haptoglobin**	GCTATGTGGAGCACTTGGTTC	CACCCATTGCTTCTCGTCGTT
**Anxa2**	ATGTCTACTGTCCACGAAATCCT	CGAAGTTGGTGTAGGGTTTGACT
**Complement factor D**	CATGCTCGGCCCTACATGG	CACAGAGTCGTCATCCGTCAC
**Taldo1**	GTAAAGCGCCAGAGGATGGAG	CTCTTGGTAGGCAGGCATCT
**Ccl9**	CCCTCTCCTTCCTCATTCTTACA	AGTCTTGAAAGCCCATGTGAAA
**Ccl21a**	GTGATGGAGGGGGTCAGGA	GGGATGGGACAGCCTAAACT
**C1qtnf9**	TGGACGAGATGGTGCCAAG	ATGTTTCCCCGGAGATCCTCT
**MMP9**	CTGGACAGCCAGACACTAAAG	CTCGCGGCAAGTCTTCAGAG
**MMP28**	AACCAGAGGTCCTAAATACTGCC	GGACGAGGCTCTACAGTGATG
**Dpt**	TGGATGGGTGAATCTTAACCGC	TCAGAGCCTTCCTTCTTGCTA
**BMP3**	ACTCCGTGAGACTGAGCCAA	CCTGTCATAGAGCCACAGCATA
**Mstn**	AGTGGATCTAAATGAGGGCAGT	GTTTCCAGGCGCAGCTTAC
**Col 1a2**	GTAACTTCGTGCCTAGCAACA	CCTTTGTCAGAATACTGAGCAGC
**Col 4a2**	GACCGAGTGCGGTTCAAAG	CGCAGGGCACATCCAACTT
**Col 5a2**	ACAGGTGAAGTGGGATTCTCA	CCATAGCACCCATTGGACCA
**Col 6a1**	CTGCTGCTACAAGCCTGCT	CCCCATAAGGTTTCAGCCTCA
**Col 8a1**	ACTCTGTCAGACTCATTCAGGC	CAAAGGCATGTGAGGGACTTG
**Loxl1**	GAGTGCTATTGCGCTTCCC	GGTTGCCGAAGTCACAGGT
**Fbln5**	GCTTGTCGTGGGGACATGAT	TGGGGTAGTTGGAAGCTGGTA
**Cyr61**	CTGCGCTAAACAACTCAACGA	GCAGATCCCTTTCAGAGCGG
**Rarres2**	GCCTGGCCTGCATTAAAATGG	CTTGCTTCAGAATTGGGCAGT
**Sox2**	GCGGAGTGGAAACTTTTGTCC	CGGGAAGCGTGTACTTATCCTT
**Nannog**	TCTTCCTGGTCCCCACAGTTT	GCAAGAATAGTTCTCGGGATGAA
**PPARγ**	TCGCTGATGCACTGCCTATG	GAGAGGTCCACAGAGCTGATT

Reference

1. Gao Y*, et al.* (2017) TNFalpha-YAP/p65-HK2 axis mediates breast cancer cell migration. *Oncogenesis* 6(9):e383.
2. Wrede CE, Dickson LM, Lingohr MK, Briaud I, & Rhodes CJ (2002) Protein kinase B/Akt prevents fatty acid-induced apoptosis in pancreatic beta-cells (INS-1). *The Journal of biological chemistry* 277(51):49676-49684.
3. Cha SH, Hwang Y, Kim KN, & Jun HS (2018) Palmitate induces nitric oxide production and inflammatory cytokine expression in zebrafish. *Fish & shellfish immunology* 79:163-167.
4. Sinha S, Perdomo G, Brown NF, & O'Doherty RM (2004) Fatty acid-induced insulin resistance in L6 myotubes is prevented by inhibition of activation and nuclear localization of nuclear factor kappa B. *The Journal of biological chemistry* 279(40):41294-41301.
5. Ruan H, Zarnowski MJ, Cushman SW, & Lodish HF (2003) Standard isolation of primary adipose cells from mouse epididymal fat pads induces inflammatory mediators and down-regulates adipocyte genes. *The Journal of biological chemistry* 278(48):47585-47593.
6. Wetzels S*, et al.* (2018) Characterization of Immune Cells in Human Adipose Tissue by Using Flow Cytometry. *Journal of visualized experiments : JoVE* (133).
7. Du C*, et al.* (2014) MTDH mediates trastuzumab resistance in HER2 positive breast cancer by decreasing PTEN expression through an NFkappaB-dependent pathway. *BMC cancer* 14:869.
